# Transfected plasmid DNA is incorporated into the nucleus via nuclear envelope reformation at telophase

**DOI:** 10.1038/s42003-022-03021-8

**Published:** 2022-01-20

**Authors:** Tokuko Haraguchi, Takako Koujin, Tomoko Shindo, Şükriye Bilir, Hiroko Osakada, Kohei Nishimura, Yasuhiro Hirano, Haruhiko Asakawa, Chie Mori, Shouhei Kobayashi, Yasushi Okada, Yuji Chikashige, Tatsuo Fukagawa, Shinsuke Shibata, Yasushi Hiraoka

**Affiliations:** 1 Graduate School of Frontier Biosciences, Osaka University, 1-3 Yamadaoka, Suita, 565-0871 Japan; 2 Advanced ICT Research Institute Kobe, National Institute of Information and Communications Technology, 588-2 Iwaoka, Iwaoka-cho, Nishi-ku, Kobe, 651-2492 Japan; 3 Keio University, School of Medicine, Shinjuku-ku, Tokyo, 160-8582 Japan; 4 RIKEN Center for Biosystems Dynamics Research (BDR), Suita, Osaka 565-0874 Japan; 5 Department of Cell Biology, Department of Physics, Universal Biology Institute (UBI) and International Research Center for Neurointelligence (IRCN), the University of Tokyo, Tokyo, 113-0033 Japan

**Keywords:** Nuclear envelope, Cellular imaging

## Abstract

DNA transfection is an important technology in life sciences, wherein nuclear entry of DNA is necessary to express exogenous DNA. Non-viral vectors and their transfection reagents are useful as safe transfection tools. However, they have no effect on the transfection of non-proliferating cells, the reason for which is not well understood. This study elucidates the mechanism through which transfected DNA enters the nucleus for gene expression. To monitor the behavior of transfected DNA, we introduce plasmid bearing *lacO* repeats and RFP-coding sequences into cells expressing GFP-LacI and observe plasmid behavior and RFP expression in living cells. RFP expression appears only after mitosis. Electron microscopy reveals that plasmids are wrapped with nuclear envelope (NE)‒like membranes or associated with chromosomes at telophase. The depletion of BAF, which is involved in NE reformation, delays plasmid RFP expression. These results suggest that transfected DNA is incorporated into the nucleus during NE reformation at telophase.

## Abstract (web summary)

Haraguchi et al. investigate how transfected DNA is incorporated into the nucleus using light and electron microscopy, and the LacI/LacO system. The authors report that cytoplasmic localised plasmid DNA is incorporated into the nucleus as it reforms during M-exit and transgene expression was achieved only after nuclear reformation in the next G1.

## Introduction

Transfection that introduces DNA plasmids into cells is an essential technology in the life sciences and medical fields, such as in gene therapy. Non-viral vectors and transfection reagents, including cationic lipids or non-liposomal lipids, have been developed as safe tools for introducing exogenous DNA into cells. DNA transfected with these reagents is incorporated into the endosome via endocytosis and exposed to the cytosol upon endosome rupture^1–5^, where it is detected by DNA sensor molecules^6–10^. However, it remains unclear how the transfected DNA in the cytosol enters the nucleus. It is empirically known that DNA transfection, which leads to gene expression from foreign DNA, is effective in proliferating cells but not in non-proliferating cells^11–13^. Therefore, it is considered that DNA enters the nucleus during mitosis when the nuclear envelope (NE) breaks down^14–16^. However, how NE breakdown is involved in the nuclear entry of exogenous DNA remains to be elucidated.

Several attempts have been made to visualize the behavior of transfected DNA and cellular responses to it^8,17,18^. These include methods using fluorescent dye-conjugated DNA^17^, *lacO*/GFP-LacI visualizing systems^17^, fluorescent nanodiamond particles coated with DNA^18^, and beads conjugated with plasmid DNA (DNA-beads)^8^. In the DNA-bead method, it has been demonstrated that DNA-beads are internalized into cells via endocytosis^8,19^, entrapped in endosomes, and exposed to the cytosol when the endosome ruptures^8^. Endosome rupture stimulates autophagosome formation in the region of the transfected DNA in a p62-dependent manner^20^, leading to its degradation. However, DNA on the beads simultaneously induces NE formation around the DNA-bead in a barrier-to-autointegration factor (BAF)-dependent manner^8^. When NE forms around the DNA beads, autophagy is suppressed^8^.

BAF is a DNA-binding protein^21–24^ involved in NE reformation at the telophase of mitosis^25–27^. It binds to foreign DNA, such as transfected DNA and viral DNA, in the cytosol^8,21,28,29^, where it assembles the NE-like membrane containing LEM domain proteins, such as emerin, around the transfected DNA^8^. This BAF-dependent NE assembly is considered to occur competitively with the assembly of the autophagy membrane^8,30^. However, the biological significance of NE assembly in transfected DNA present in the cytosol is unclear.

NE is a double-membrane structure comprising the inner and outer nuclear membranes. The outer membrane is connected to the endoplasmic reticulum (ER) and contains proteins shared with the ER, whereas the inner membrane contains transmembrane proteins specific to the NE. Among the inner nuclear membrane proteins, LEM domain NE proteins, including lamina-associated polypeptide 2 (Lap2), emerin, MAN1, and Lem2, are highly conserved among metazoans^31,32^. LEM domain proteins bind BAF with the LEM domain^25,33–35^. Beneath the inner nuclear membrane, there is a nuclear lamina consisting of lamins^36,37^. There are fenestrated structures, called nuclear pores, spanning the double-membrane structure of the NE^38,39^. The nuclear pore complex (NPC) is formed in the pores, and nucleocytoplasmic transport between the nucleus and the cytoplasm is carried out through the NPC.

The NE is disassembled at the beginning of mitosis (prometaphase) and reforms around chromosomes at the end of mitosis (telophase) in higher eukaryotic cells, including human cells. During NE reformation at telophase, emerin and other LEM domain proteins assemble at the “core” region of the chromosome mass in a BAF-dependent manner, whereas lamin B receptor (LBR) and NPC components assemble in the “non-core” region in a BAF-independent manner^25,27,40–42^. The ESCRT-III protein complex seals the gaps in the nuclear membranes during NE reformation at telophase^43,44^.

To elucidate when and how transfected exogenous DNA enters the nucleus, we transfected cells with DNA plasmids designed to visualize plasmids and monitor their expression. Herein, we show that transfected DNA is incorporated into the nucleus when the NE is reformed during telophase. Our findings will contribute to understanding how foreign DNA behaves within cells, providing useful information for generating improved transfection reagents and techniques.

## Results

### Protein expression from exogenous DNA occurs only after mitosis

**Fig. 1:**
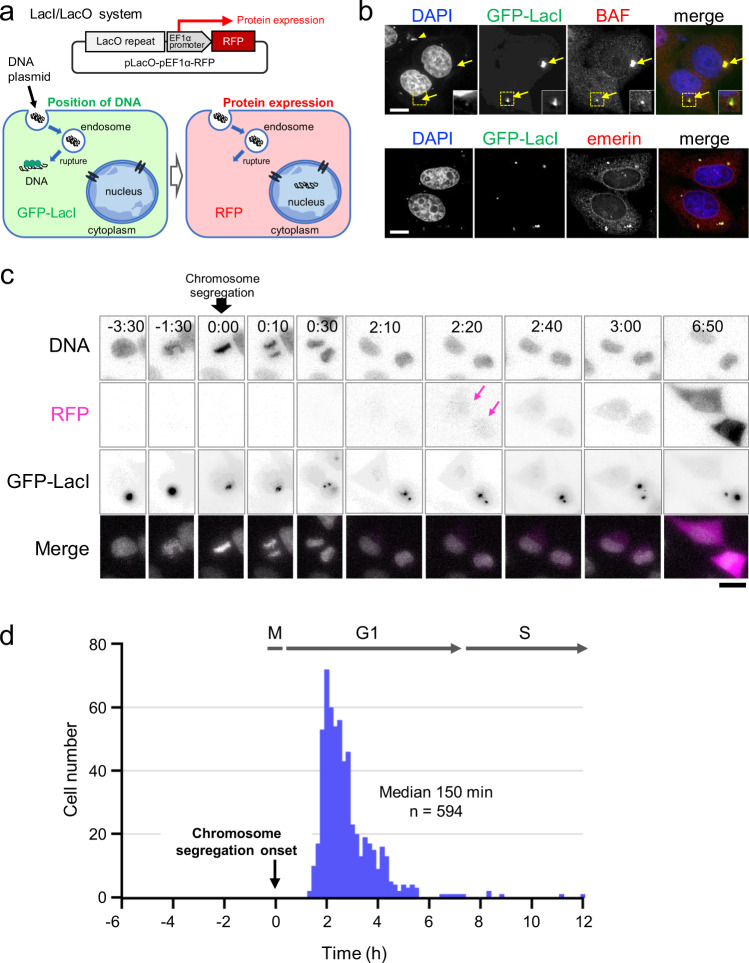
Gene expression from foreign DNA occurs after mitosis. **a** Schematic of the experiment. Top: diagram of pLacO-pEF1α-RFP. This plasmid carries a *lacO* repeat sequence (256 repeats, approximately 10 kbp) and a sequence expressing a monomeric red fluorescent protein (RFP) under the EF1α promoter. Bottom left: when the transfected plasmid is exposed to the cytosol upon endosome rupture, it binds to GFP-LacI and becomes fluorescent. Bottom right: when it enters the nucleus, the expression of RFP occurs. Therefore, RFP fluorescence is an indication of the nuclear translocation of the plasmid. While using this system, exogenous gene expression was determined by the appearance of RFP fluorescence. **b** HeLa/GFP-LacI cells transfected with pLacO-pEF1α-RFP (0.5 μg/dish) diagrammed in **a**. Immediately after transfection, the cells were fixed and stained using anti-BAF or anti-emerin antibodies. DNA (stained with DAPI, blue), GFP-LacI (green), and BAF (red) or emerin (red) in the merged image. Arrows indicate DAPI signals with GFP-LacI signals. Arrowhead indicates DAPI signals without GFP-LacI signals. The inset shows an image with increased brightness in the boxed region. Bar, 10 μm. **c** Time-lapse live-cell images of transfected HeLa/GFP-LacI cells. DNA was stained with Hoechst 33342 to determine the timing of chromosome segregation. Immediately after transfection, acquisition of time-lapse images at 10-min intervals was started. Selected time frames are shown. Images at each time frame are maximum intensity projection images for *z*-stacks. The numbers represent time (h:min). Time 0 represents the onset of chromosome segregation. Merged images, DNA (Hoechst 33342, white) and RFP (magenta). Bar, 20 μm. **d** Timing of protein expression from transfected DNA. The timings of RFP expression and chromosome segregation were determined in individual cells by time-lapse live-cell imaging as described in **c**. *X* axis represents the time of RFP expression in hours when the timing of chromosome segregation was set to zero for each cell. *Y* axis is the number of cells that expressed RFP at that time. Cell numbers plotted were *n* = 248 by DV and *n* = 346 by LSM880; total number, *n* = 594. These data are combined in this graph.

To visualize transfected DNA and its expression, we generated the following experimental system. We constructed a non-viral DNA plasmid carrying a *lacO* repeat sequence (256 repeats, approximately 10 kbp) and a sequence expressing monomeric red fluorescent protein (RFP) driven by the EF1α promoter (pLacO-pEF1α-RFP, Fig. 1a). We introduced it into human HeLa cells stably expressing EGFP-LacI (hereafter HeLa/GFP-LacI cells) using a non-liposomal lipid transfection reagent. Thus, when the DNA plasmid enters the cytosol after endosome rupture, it is visualized by binding to GFP-LacI, forming fluorescent puncta that are easily detected using fluorescence microscopy (Fig. 1a; arrows, Fig. 1b). Plasmid retained in the endosome cannot bind GFP-LacI and therefore will not be fluorescent (arrowhead, Fig. 1b).

We first tested the efficacy of the GFP-LacI signal in detecting transfected exogenous DNA in the cytosol. After the removal of the transfection reagents, the cells were fixed and subjected to indirect immunofluorescence staining using anti-BAF. We examined the overlap between the GFP-LacI signal and BAF, because BAF is known to bind exogenous DNA immediately after it is released into the cytosol by endosome rupture^8,20,30^. We found that the cytoplasmic GFP-LacI puncta overlapped with BAF (Fig. 1b), indicating that our system visualized cytosolic plasmid DNA. We also used the DNA-specific fluorescent dye, 4′,6-diamidino-2-phenylindole (DAPI), to confirm that the puncta contained DNA. Some cytoplasmic DAPI puncta did not colocalize with the GFP-LacI signal (see arrowhead, Fig. 1b), suggesting that some plasmids were retained in endosomes. The GFP-LacI-positive puncta colocalized with the LEM domain NE protein emerin (lower panels, Fig. 1b and Supplementary Fig. 1a, b) and Lem2 (Supplementary Fig. 1a, b). These puncta were also colocalized with LBR and calnexin (CNX) but not with lamin B1 and early endosome antigen 1 (EEA1) (Supplementary Fig. 1a, b). These results suggested that they were surrounded by an NE-like membrane that originated from the ER membrane in the cytosol after being released from the endosome, which is consistent with a report demonstrating that DNA-bead complexes become wrapped with NE-like membranes within 5–10 min after endosome rupture^8^.

Subsequently, to determine the timing of protein expression from transfected DNA, HeLa/GFP-LacI cells were transfected with the pLacO-pEF1α-RFP plasmid for 4 h, and RFP expression from the plasmid in individual cells was monitored by time-lapse live-cell imaging using a fluorescence microscope. Time-lapse live-cell observation started immediately after transfection. DNA was stained with Hoechst 33342 during transfection, and DNA staining was used to monitor chromosome segregation during mitosis. Images were obtained every 10 min for at least 18 h using a DeltaVision or LSM880 microscope. A representative example of live-cell imaging is shown in Fig. 1c (Supplementary Movies 1 and 2). In this case, the timing of RFP expression was 140 min after the onset of chromosome segregation. We repeated the live-cell imaging analysis for more than 600 individual cells. Among the 662 cells analyzed, 594 cells expressed RFP, and the remaining 68 cells did not express detectable amounts of RFP within 18 h of observation. The timing of the appearance of the RFP signal in the individual cells was plotted with the timing of chromosome segregation as zero (Fig. 1d). The results showed that RFP signals appeared only in cells that had proceeded through mitosis (total number of cells plotted, *n* = 594, Fig. 1d). None of the cells expressed RFP before mitosis under these conditions. This result suggests that protein expression from the transfected plasmid did not occur during the interphase before mitosis. RFP expression was detected 2–5 h after the onset of mitotic chromosome segregation (median 150 min, *n* = 594) (Fig. 1d). As the G1 phase lasts for 6–8 h in the HeLa cell line^45^, these results suggest that protein expression occurs during the G1 phase in most cells. In these cells, the RFP signal appeared to be comparable between the two daughter cells. However, in a small fraction of the cells, RFP fluorescence appeared later, after 500 min (6 h 20 min). In these cells, the RFP signal was often confined to only one daughter cell, the reason for which remains unknown.

To confirm the importance of mitosis for protein expression from transfected DNA, we arrested the cells at or before the mitotic phase (M-phase) using nocodazole, a microtubule depolymerization drug. HeLa/GFP-LacI cells were transfected with the pLacO-pEF1α-RFP plasmid and incubated with the culture medium in the presence or absence of nocodazole for 18 h. In the control cells incubated without nocodazole, RFP expression was observed in many cells (control in Supplementary Fig. 1c). In contrast, in cells incubated with nocodazole, RFP expression was greatly suppressed (+Noc 18 h in Supplementary Fig. 1c), and recovered by the removal of nocodazole (release in Supplementary Fig. 1c). To verify the expression of RFP, the amount of RFP was evaluated by western blotting (WB) analysis using anti-RFP antibody (Supplementary Fig. 1d). The results showed that RFP expression was greatly suppressed to an undetectable level in the M-phase-arrested cells with nocodazole compared with the expression in the control cells but was recovered by the removal of the drug (Anti-RFP in Supplementary Fig. 1d). These results suggest that a mitosis is an important event for protein expression from transfected foreign DNA.

We also investigated protein expression from transfected DNA in U2OS cells, a human osteosarcoma-derived cell line. U2OS cells were transfected with the pEF1α-RFP plasmid, and RFP expression from the plasmid in individual cells was monitored by time-lapse live-cell imaging using a fluorescence microscope. DNA was stained with Hoechst 33342 to determine the timing of chromosome segregation during mitosis. Among the 113 cells observed, 107 cells expressed RFP, whereas the remaining six cells did not. The timing of RFP expression in individual cells was plotted in a graph with the timing of chromosome segregation as zero (*n* = 107, Supplementary Fig. 1e). The results showed that RFP expression did not occur before mitosis in U2OS cells, similar to HeLa cells. The timing of RFP expression in U2OS cells was 2–5 h (median 150 min) after the onset of chromosome segregation (Supplementary Fig. 1e). This median value was the same as that obtained for HeLa cells. These results indicate that protein expression from exogenous DNA transfected using a non-viral transfection reagent occurs only after mitosis. Our findings provide clear evidence that non-viral transfection is effective only for proliferating cells.

### Dynamic behavior of exogenous DNA during mitosis

**Fig. 2:**
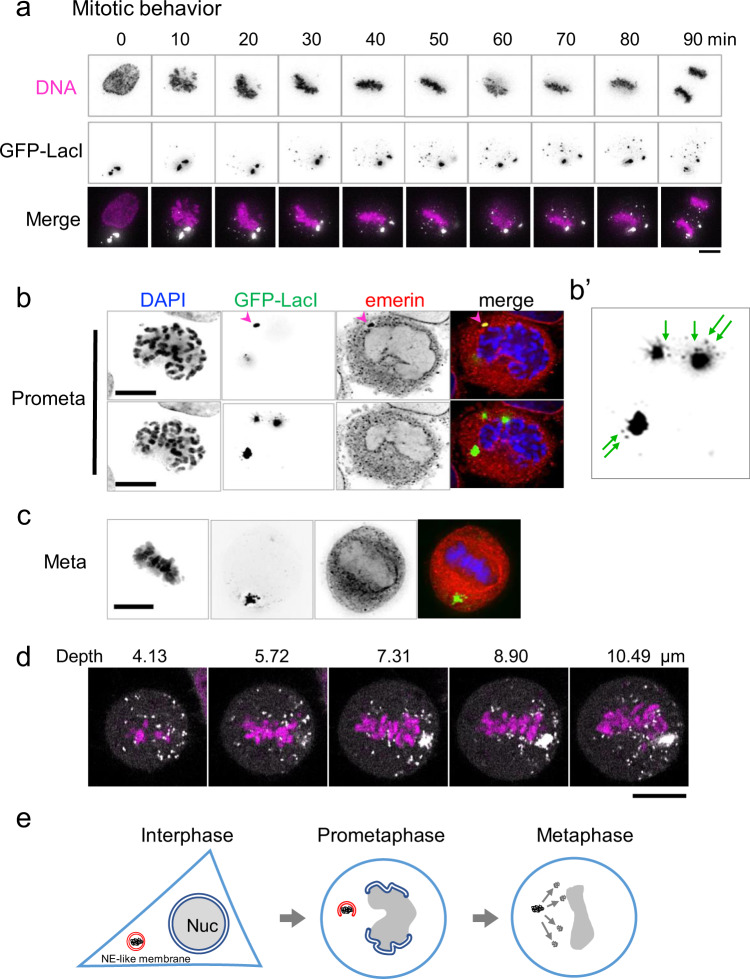
DNA puncta disperse during mitosis. **a** Representative time-lapse images of HeLa/GFP-LacI cells transfected with pLacO-pEF1α-RFP. DNA was stained with Hoechst 33342. Immediately after transfection, acquisition of time-lapse images at 10-min intervals was started. Time 0 represents the leftmost image (680 min after the start of image acquisition). DNA (magenta) and GFP-LacI (white) in the merged images. Bar, 10 μm. **b** HeLa/GFP-LacI cells transfected with pLacO-pEF1α-RFP, fixed, and stained for emerin as indicated in Fig. 1b. A single, representative prometaphase cell is shown. The upper and lower rows show the same cell at focal planes 1.5 μm apart. Arrowhead indicate puncta with emerin signals. DAPI (DNA, blue), GFP-LacI (green), and emerin (red) are shown in the merged image. Bars, 10 μm. **b′** Enlarged image of the lower panel of GFP-LacI in Fig. 2b. Arrows indicate small puncta. **c** A metaphase cell is shown in the same manner as in **b**. Bar, 10 μm. **d** Super-resolution images of GFP-LacI and chromosomes in a metaphase cell. HeLa/GFP-LacI cells were transfected with pLacO-pEF1α-RFP. After transfection, cells were fixed with a fixative as described in Methods. Super-resolution images were obtained using LSM880 Airyscan microscopy. White and magenta represent small puncta of transfected DNA (GFP-LacI) and chromosomes (DAPI), respectively. Bar, 10 μm. **e** Diagrams of transfected DNA in interphase, prometaphase, and metaphase cells.

Protein expression from plasmid DNA did not occur in some cells, even in the presence of GFP-LacI-positive clusters. To understand the difference in the behavior of plasmid DNA between cells with and without protein expression, we characterized the dynamic behavior of GFP-LacI clusters during mitosis. Time-lapse analysis of GFP-LacI in mitotic cells revealed that in most cells, GFP-LacI clusters were disjointed at prometaphase and dispersed into smaller puncta during mitosis (Fig. 2a and Supplementary Movies 1, 3). In contrast, in some cells, GFP-LacI clusters remained intact during mitosis. Notably, most cells with GFP-LacI puncta dispersed during mitosis expressed RFP during the subsequent interphase (62 out of 68 cells observed) (Supplementary Fig. 2a), whereas the cells without dispersion did not (6 out of 68 cells) (Supplementary Fig. 2b), suggesting that the dispersion of plasmids may be important for protein expression in the subsequent interphase.

We then investigated whether the presence or absence of NE was involved in the GFP-LacI dispersion. Indirect immunofluorescence staining revealed that GFP-LacI puncta colocalized with emerin in interphase cells (lower panels, Fig. 1b). In prometaphase cells, in which NE breakdown is ongoing, some of the GFP-LacI puncta colocalized with emerin (arrowhead, Fig. 2b), whereas others did not (lower panels, Fig. 2b). Notably, small puncta were observed near the larger puncta with no NE (arrows, Fig. 2b′ and Supplementary Fig. 2c), indicating that dispersion occurred upon NE breakdown. In metaphase cells, GFP-LacI puncta did not colocalize with emerin (Fig. 2c). Many small puncta of GFP-LacI of the same size were typically found in metaphase cells (white puncta, Fig. 2d and Supplementary Movie 4). These results suggest that the dispersion of GFP-LacI puncta during mitosis is caused by the breakdown of the NE-like membrane surrounding the puncta. Figure 2e summarizes the behavior of GFP-LacI puncta during mitosis.

### Behaviors of plasmid DNA during NE reformation at telophase

**Fig. 3:**
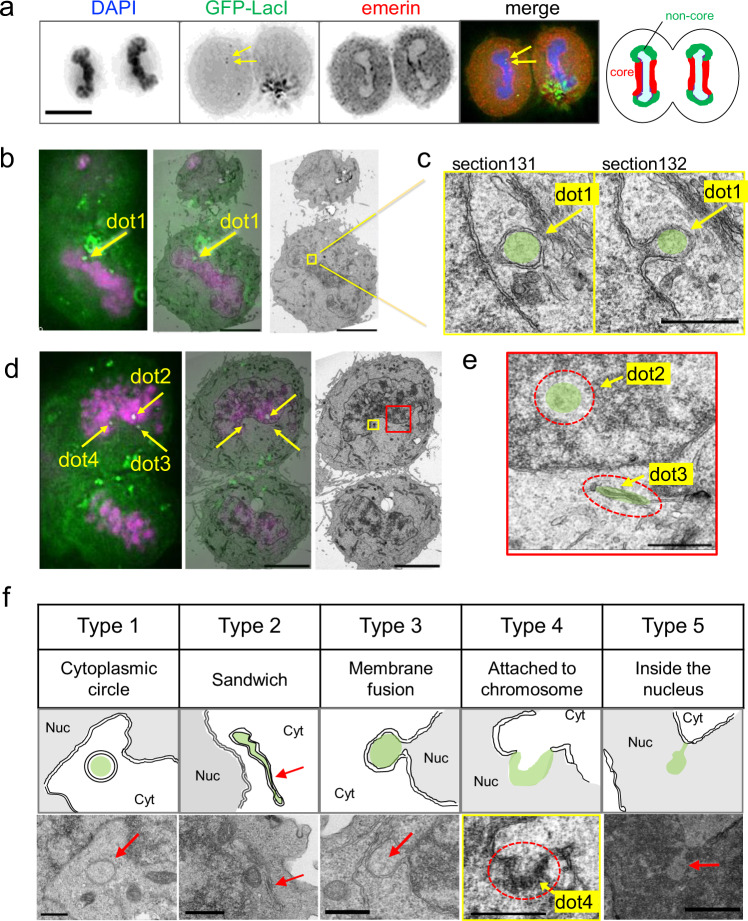
CLEM images showing small puncta of transfected DNA in telophase cells. HeLa/GFP-LacI cells were transfected with pLacO-pEF1α-RFP, and then fixed and subjected to indirect immunofluorescence imaging (**a**) or CLEM (**b**–**f**). **a** Indirect immunofluorescence images of telophase cells. After the removal of the transfection reagent, the cells were fixed and immunostained for emerin. DAPI (DNA, blue), GFP-LacI (green), and emerin (red) are shown in the merged image. The rightmost cartoon illustrates the “core” (red) and “non-core” (green) regions of the NE, forming around the telophase chromosome mass. Bars, 10 μm. **b** Representative CLEM images of telophase cells. Left, fluorescence microscopic image; middle, merged image; right, electron microscopic image. Colors represent GFP-LacI (green) and DAPI (magenta). Arrows indicate the position of GFP-LacI (dot 1). Bar, 5 μm. **c** Higher magnification of the yellow-boxed region in **b**. Arrows indicate the positions of GFP-LacI (dot 1, shaded in green). Section131 and section132 are the neighboring sections, 80 nm apart. Bar, 500 nm. **d** Different focal planes of the cell shown in **b**. Left, fluorescence microscopic image; middle, merged image; right, electron microscopic image. Colors represent GFP-LacI (green) and DAPI (magenta). Arrows, positions of GFP-LacI (dots 2–4). Bar, 5 μm. **e** Higher magnification of the red-boxed region in **d**. Red circles represent the regions of the fluorescence signals, shaded in green. Bar, 500 nm. **f** Classification of puncta morphologies. Type 1 (cytoplasmic circle): plasmid contained within a circular (or spherical) structure surrounded by an NE-like membrane that is present in the cytoplasm. Type 2 (sandwich): plasmid is present between NE-like membranes. Type 3 (membrane fusion): plasmid contained in the cytoplasmic circle fused with the NE. Type 4 (attached to chromosome): plasmid attached to the telophase chromosomes. Dot 4 is the same dot 4 shown in **d**. Type 5 (inside the nucleus): plasmid inside the nucleus. Middle panels, schematics of the electron microscopic images. NE is shown as a double line. Red arrows indicate the positions of GFP-LacI, shaded in green. Electron microscopic images of wider areas, including the area shown in this figure, are shown in Supplementary Fig. 3. Bars, 500 nm for Types 1-4 and 1 μm for Type 5.

As protein expression occurs only after mitosis (Fig. 1c, d and Supplementary Fig. 1c–e), we hypothesized that NE reformation at the end of mitosis might be important for the nuclear entry of the plasmid DNA. In fact, small puncta of GFP-LacI, probably containing a single copy or a few copies of plasmid DNA, were frequently observed near the telophase chromosomes (arrows, Fig. 3a). To visualize the structural details of these GFP-LacI puncta, we performed correlative light and electron microscopy (CLEM) on telophase cells undergoing NE reformation (Fig. 3b–f and Supplementary Fig. 3). We selected telophase cells approximately 10 min after the onset of chromosome segregation. In these cells, the NE is assembled at the “non-core” region of the chromosome mass whereas not yet at the “core” region (right drawing in Fig. 3a)^27^. LEM domain proteins assemble at the “core” region in a BAF-dependent manner, whereas LBR and the NPC assemble at the “non-core” region in a BAF-independent manner^25,27,42^.

CLEM revealed that puncta near the chromosomes at telophase were surrounded by a double-membrane structure similar to the NE or ER (labeled dot 1 in Fig. 3b, c). This NE-like membrane appeared to be fused with the NE on the chromosome (Fig. 3c), suggesting that the transfected DNA may enter the nucleus via membrane fusion. Multiple additional structures were found: dot 2 in Fig. 3d was inside the nucleus (Fig. 3e), whereas dot 3 was outside and sandwiched between two sets of ER or NE precursor membranes (Fig. 3e). Dot 4 in Fig. 3d was attached to the chromosomes (Type 4 in Fig. 3f and Supplementary Fig. 3l–o). In addition, the relatively larger puncta located in the cytoplasm were surrounded by ER or NE precursor membranes (Type 1 in Fig. 3f and Supplementary Fig. 3a–d). We classified these puncta into five types based on their morphologies obtained by CLEM, as indicated in Fig. 3f and Supplementary Fig. 3: Type 1 (cytoplasmic circle) is a relatively large circular structure covered with a double membrane, which is often observed around the relatively large GFP-LacI puncta present in the cytoplasm. Type 2 (sandwich) is an electron-dense structure squeezed between two double membranes, suggestive of ER or NE precursor membranes (Fig. 3e, f and Supplementary Fig. 3e–g). Type 3 (membrane fusion) is a structure in which a relatively large circular structure covered with a double membrane appears to be fused to the NE (Fig. 3c, f and Supplementary Fig. 3h–k). Type 4 (attached to chromosome) is a structure at the edge of the chromosomal region where the NE is not present (Fig. 3f and Supplementary Fig. 3l–o). Type 5 (inside the nucleus) is a structure inside the nucleus (between chromosomes) (Fig. 3f and Supplementary Fig. 3p–s). In addition to these types, we frequently observed large GFP-LacI puncta in the cytoplasm. These puncta were found in degradative organelles, including lysosomes and autophagosomes.

**Fig. 4:**
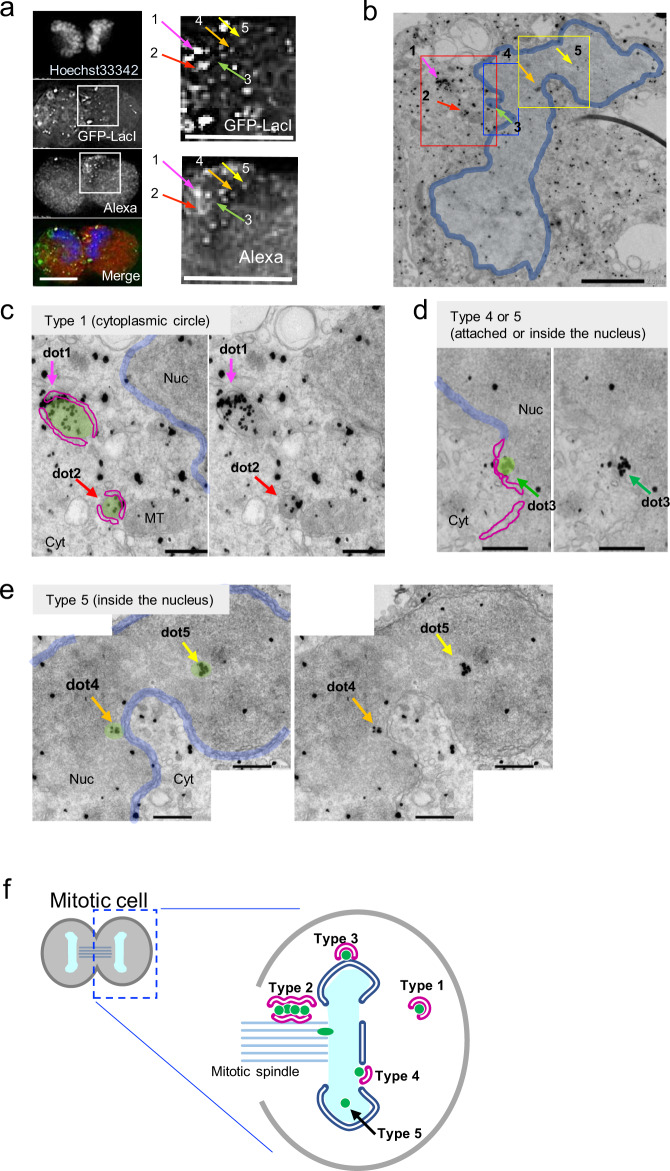
Immuno-CLEM (iCLEM) images of telophase cells. HeLa/GFP-LacI cells were transfected with pLacO-pEF1α-RFP, and then fixed and subjected to iCLEM. **a** Fluorescence microscopic images. Left: Overview fluorescence images. DNA (stained with Hoechst 33342, blue), GFP-LacI (green), and Alexa-nanogold (red) in the merged image. Right: Enlarged images of the white-boxed region in the left panel. Numbers 1–5 correspond to the dots analyzed in **b**–**e**. Bars, 10 μm. **b** Low-magnification electron micrograph of the telophase cell shown in **a**. Blue contour, the edge of telophase chromosome mass. Arrows indicate the positions analyzed. Red-, blue-, and yellow-boxed regions are enlarged in **c**–**e**, respectively. Bar, 2 μm. **c** Representative type 1, from the red-boxed region in **b**. Red contour, NE-like structures. Blue contour, edge of the nucleus. Arrows indicate the positions of dots 1 and 2. Nuc, Cyt, and MT label the nucleus, cytoplasm, and mitochondria, respectively. Bars, 500 nm. **d** Representative type 4 or 5, from the blue-boxed region in **b**. Red contour, NE-like structures. Blue contour, edge of the nucleus. Arrows indicate the positions of dot 3. Nuc and Cyt label the nucleus and cytoplasm, respectively. Bars, 500 nm. **e** Representative type 5, from the yellow-boxed region in **b**. Blue contour, edge of the nucleus. Arrows indicate the positions of dots 4 and 5. Nuc and Cyt label the nucleus and cytoplasm, respectively. Bars, 500 nm. **f** Diagrams of typical localizations of transfected DNA in telophase cells.

To confirm that the region allocated by CLEM contained the GFP-LacI signal, immuno-CLEM analysis was performed using an anti-GFP antibody (Fig. 4). Several GFP-LacI puncta localized near the telophase chromosome mass were examined (Fig. 4a–e). Target dots 1 and 2 in Fig. 4a, b were located inside a relatively large circle surrounded by a double-membrane structure similar to NE or ER (Fig. 4c); therefore, they were classified as type 1. Target dot 3 was located on the edge or inside of the chromosomal region and classified as type 4 or 5 (Fig. 4d and Supplementary Fig. 4). Target dots 4 and 5 were located inside the nucleus and were classified as type 5 (Fig. 4e and Supplementary Fig. 4). These results suggest that at least part of the transfected DNA was attached to the telophase chromosome or located in the nucleus.

Taken together, these results suggest that the transfected DNA can be incorporated into the nucleus during NE reformation at telophase during mitosis. Figure 4f shows the typical localization of GFP-LacI puncta at the telophase.

### BAF-dependent NE reformation at telophase facilitates nuclear entry

To understand the molecular mechanism underlying the nuclear entry of exogenous DNA in telophase, we depleted NE proteins and examined the effect on protein expression from the transfected DNA. BAF, emerin, and Lem2 were selected as target proteins for depletion because they are all known to be involved in NE reformation during telophase^27,46–48^. We first employed the auxin-inducible degron (AID)-induced degradation system to specifically deplete a target protein at the time of interest, for example, only during mitosis^49,50^. Therefore, we integrated a sequence of mClover3-mAID into each of the three target genes (*BAFN1*, *EMD*, and *LEMD2*) using CRISPR/Cas9 editing^51^. We successfully produced *LEMD2* mutant cell lines (HeLa/mClover3-mAID-Lem2) expressing an mClover3-mAid-Lem2 protein as the sole Lem2 protein (Supplementary Fig. 5a). WB of lysates of cells treated with indole-3-acetic acid (IAA) for 60 min revealed that Lem2 was almost completely lost (Supplementary Fig. 5b), consistent with the time-lapse microscopy data (Supplementary Fig. 5c and Supplementary Movie 5). However, attempts to produce *BAFN1* and *EMD* mutant cell lines have been unsuccessful.

**Fig. 5:**
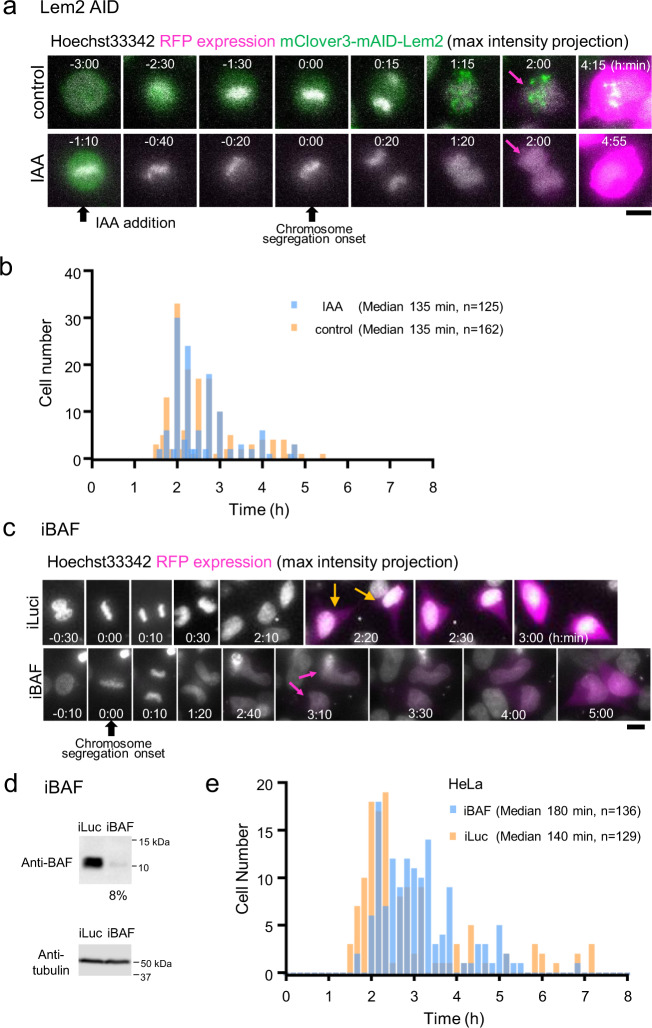
Depletion of BAF delays protein expression from exogenous DNA. **a** Time-lapse images of HeLa/mClover3-mAID-Lem2 cells. HeLa/mClover3-mAID-Lem2 were transfected with pEF1α-RFP plasmid. DNA was stained with Hoechst 33342 to determine the timing of chromosome segregation. Immediately after transfection, time-lapse live-cell images were acquired every 10 or 15 min (2 μm × 5 *z*-stacks for each time point). IAA (indole-3-acetic acid) or ethanol as a control was added to the cells during live-cell imaging. Maximum intensity projection images of selected time points are shown. Colors represent mClover-mAID-Lem2 (green), RFP (magenta) and Hoechst 33342 (white). The numbers represent time (h:min). Black arrows, timings of the addition of IAA and the onset of chromosome segregation as indicated. Magenta arrows, timing of RFP expression. Bar, 10 μm. **b** Timing of RFP expression in Lem2-depleted HeLa (IAA) and control parental (control) cells. The timing of RFP expression in individual cells was determined by time-lapse imaging of each cell as shown in **a**. *X* axis represents the time of RFP expression in hours when the timing of chromosome segregation was set to zero for each cell. *Y* axis is the number of cells that expressed RFP at that time. Cell numbers plotted and the median values of RFP expression are shown in the graph. **c** Time-lapse images of transfected HeLa cells treated with siRNA targeting *BAFN1* (iBAF) and control siRNA (iLuc). Cells were treated with siRNA (iBAF or iLuc) and transfected with pEF1α-RFP plasmid. DNA was stained with Hoechst 33342 to determine the timing of chromosome segregation. Immediately after transfection, time-lapse live-cell images were acquired at 10-min intervals (2 μm × 5 *z*-stacks for each time point). Images projected by the maximum intensity projection for *z*-stacks are shown. Colors represent RFP expression (magenta) and Hoechst 33342 (white). The numbers represent time (h:min). Time 0 represents the onset of chromosome segregation (black arrow). Orange and magenta arrows, timing of RFP expression. Bar, 10 μm. **d** Western blotting of lysates of HeLa cells treated with siRNA targeting *BAFN1* (iBAF) and control siRNA (iLuc). Tubulin was used as a loading control. **e** Timing of RFP expression in HeLa cells treated with iBAF and iLuc. The timing was determined by time-lapse live-cell imaging of individual cells as shown in **c**. *X* axis represents the time of RFP expression in hours when the timing of chromosome segregation was set to zero for each cell. *Y* axis is the number of cells that expressed RFP at that time. Cell numbers are plotted and the median values of RFP expression are shown in the graph.

We examined the effect of Lem2 depletion on protein expression from the transfected plasmid using the HeLa/mClover3-mAID-Lem2 cell line (Fig. 5a, b). The cells were transfected with a pEF1α-RFP plasmid, and RFP expression from the plasmid was monitored in individual living cells by time-lapse imaging, and DNA was stained with Hoechst 33342 during transfection to determine the timing of chromosome segregation. During time-lapse observation, IAA was added when the cells of interest proceeded to prometaphase or early metaphase. When IAA was added, the fluorescence signal of mClover3-mAID-Lem2 was almost completely lost during metaphase (IAA, 0:00, in Fig. 5a), suggesting that Lem2 was depleted at telophase in this cell. The timing of RFP expression from the transfected DNA was 120 min after the onset of chromosome segregation in this Lem2-depleted cell, similar to the 120 min in the control cells (Fig. 5a). Live-cell imaging was performed for more than 100 individual cells, and the timing of the appearance of the RFP signal in the individual cells was plotted with the onset of chromosome segregation as zero (Fig. 5b). The results showed that the timing of RFP expression in Lem2-depleted cells (IAA, median 135 min, *n* = 162) was unchanged from that of control cells (control, median 135 min, *n* = 125), suggesting that Lem2 does not influence the expression of exogenous DNA at telophase.

Subsequently, we used small interfering RNA (siRNA) to deplete the target proteins. HeLa cells were treated with siRNA against *BAFN1* (iBAF, Fig. 5c–e), with siRNA against luciferase used as a control (iLuc, Fig. 5c–e). WB revealed that the amount of BAF was reduced to 8% under these conditions (Fig. 5d and Supplementary Fig. 5d). Then, the BAF-depleted cells were transfected with the pEF1α-RFP plasmid, and RFP expression from the plasmid was monitored in individual cells by time-lapse live-cell imaging. DNA was stained with Hoechst 33342 to determine the onset of chromosome segregation. A representative example of time-lapse imaging is shown in Fig. 5c. The timing of RFP expression from the transfected DNA was 190 min after the onset of chromosome segregation in this BAF-depleted cell, whereas it was 140 min in the control cells (Fig. 5c). Live-cell imaging was performed for more than 100 individual cells, and the timing of RFP expression in the individual cells was plotted with the timing of chromosome segregation as zero (Fig. 5e). RFP expression in cells treated with *BAFN1* siRNA (*n* = 136) was delayed relative to that of the control cells (*n* = 129) (median 180 vs. 140 min for BAF siRNA and control siRNA, respectively, Fig. 5e). To confirm that BAF was truly depleted in the cells assayed by microscopy, a similar experiment was carried out using cells constitutively expressing GFP-BAF, producing similar results (Supplementary Fig. 6a, b). Because the depletion of BAF causes a delay in NE reformation during telophase^27^, our results suggest that NE reformation during telophase is an important step for the entry of exogenous DNA into the nucleus. We also tested the effect of emerin siRNA depletion; however, it had no effect on the timing of protein expression from exogenous DNA (Supplementary Fig. 6c–e).

### Intact NE is required for blocking foreign DNA from entering the nucleus

**Fig. 6:**
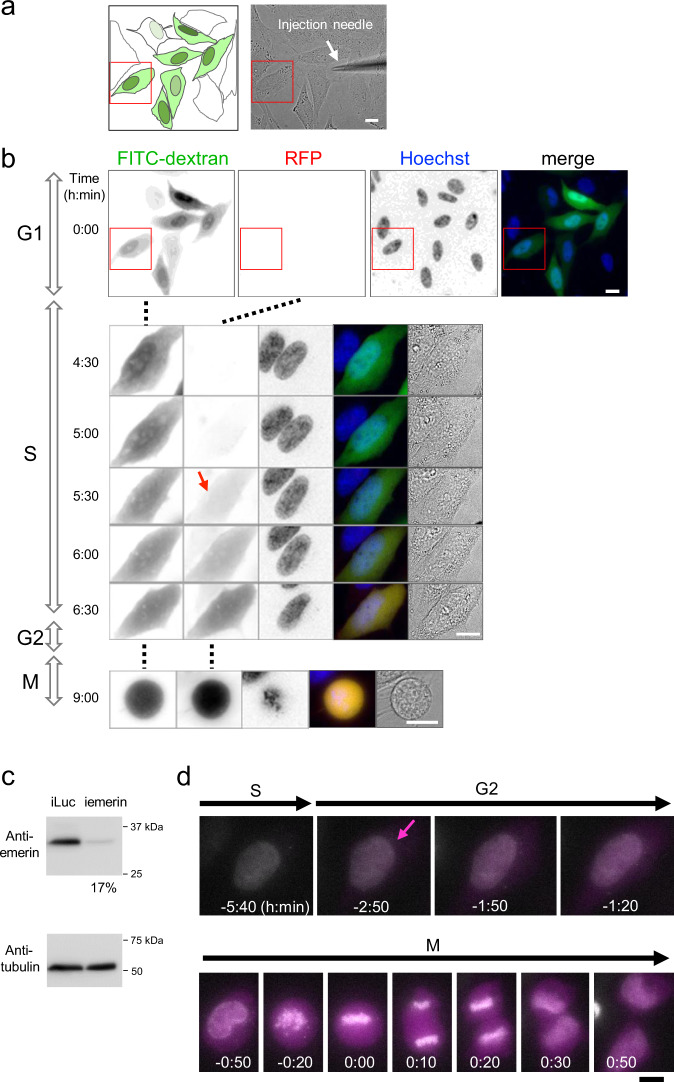
NE rupture causes premitotic gene expression. **a** HeLa cells were transfected with pEF1α-RFP plasmid. NE was ruptured with a microinjection needle by microinjecting FITC-dextran into the nucleus. Left, schematics of microinjected cells (shaded in green) in upper left panel in **b**. Regions colored in dark green represent the position of the nucleus with higher FITC-dextran signals. Right, bright-field image of the same cells as in **b**. An arrow indicates a microinjection needle used for microinjection into the nucleus to achieve NE rupture. Bar, 10 μm. **b** Time-lapse images of the microinjected cells shown in **a**. Immediately after microinjection (NE rupture), time-lapse images were acquired every 10 min (2 μm × 5 *z*-stacks for each time point). Images projected by the maximum intensity projection for *z*-stacks are shown. The cell in the red-boxed region in the top panel (time 0:00) is shown in the lower panels (time 4:30–9:00). From left to right, FITC-dextran, RFP, and Hoechst, merged, bright-field images. Colors in merged images, FITC-dextran (green), RFP (red), and Hoechst 33342 (blue). The numbers represent time (h:min) from the start of imaging. Red arrow indicates the start of RFP expression. G1, S, G2, M represent the cell-cycle phases. Bars, 10 μm. **c** Western blotting of HeLa cells treated with siRNA targeting *EMD* (iemerin) and the control (iLuc), showing that emerin expression in the cells treated with siRNA was reduced to 17% of control levels. **d** Time-lapse images of the cells treated with iemerin. HeLa cells were treated with siRNA targeting *EMD* (iemerin). The cells were transfected with pEF1α-RFP plasmid. DNA was stained with Hoechst 33342 (white). Time-lapse images were collected every 10 min (2 μm × 5 *z*-stacks for each time point) after transfection. Images projected by the maximum intensity projection for *z*-stacks are shown. Time 0 represents the onset of chromosome segregation. RFP expression (magenta) from transfected plasmid appeared in premitotic stage. Magenta arrow indicates the start of RFP expression. S, G2, and M represent the S, G2, and M phases of the cell cycle. Bar, 10 μm.

Our data suggest that intact NE in interphase cells may block the entry of foreign DNA into the nucleus. To confirm this hypothesis, we examined the effect of NE rupture on the timing of exogenous protein expression from transfected DNA. HeLa/GFP-LacI cells were transfected with the pLacO-pEF1α-RFP plasmid and microinjected with FITC-dextran into the nuclei of the selected cells to rupture their NEs physically and transiently (Fig. 6a). Then, seven cells with the FITC signal in their nuclei were subjected to time-lapse live-cell imaging to monitor RFP expression from the plasmid. Time-lapse images showed that some cells occasionally expressed RFP from plasmid DNA, even in interphase cells that had never undergone mitosis (Fig. 6b). In this example, one of the seven cells with FITC signal in their nuclei showed premitotic expression of RFP.

Similarly, premitotic protein expression from transfected DNA was observed in cells depleted of the NE protein emerin (Fig. 6c, d). When emerin was depleted with siRNA treatment (Fig. 6c and Supplementary Fig. 6d), premitotic protein expression from the transfected DNA was observed in five of the 131 cells tested, whereas it was not observed in the control siRNA cells (iLuc, *n* = 129, Supplementary Fig. 6e). A representative example of premitotic protein expression is shown in Fig. 6d. These results suggest that intact NE is required to act as a physical barrier to block the nuclear translocation of foreign DNA.

## Discussion

There exist multiple cellular barriers to the delivery of DNA plasmids to the nucleus, including intracellular uptake, endosome acidification, autophagy, immune sensing pathways, and nuclear entry^16,52,53^. The NE acts as a barrier to foreign DNA entering the nucleus^16,53–55^.

In this study, we observed the entire process from transfection to protein expression over time in individual living cells (Fig. 1c, d and Supplementary Fig. 1e). Our results demonstrate that protein expression from the exogenous pLacO-pEF1α-RFP plasmid occurs only after mitosis but not during interphase before mitosis, at least in HeLa and U2OS cells. Our finding is consistent with previous findings that exogenous gene expression effectively occurs in dividing cells^12,54–56^. In contrast, exogenous protein expression has been reported, albeit less frequently, in non-dividing myotube cells or growth-arrested cells when the plasmid was microinjected into the cytoplasm^57,58^. In addition, examples have demonstrated that the plasmid DNA enters the nucleus by active transport through the NPC^59–61^. However, our study failed to find such an example among more than 600 cells observed by time-lapse imaging. This difference may be due to differences in the cell types, promoter sequences of the plasmid, transfection methods, observation conditions, and so on. In particular, differences in transfection methods may be an issue to be considered. The plasmid transfected with the reagents enters the cytosol through the endosome and is then enwrapped with the NE-like membrane structure after endosomal rupture (Fig. 1b and Supplementary Fig. 1a, b), as described in our previous report^8^ and others^1–5,53^. In contrast, plasmids microinjected into the cytoplasm may escape this confinement by the membrane structure, allowing the membrane-free plasmids to enter the nucleus.

The difference in plasmid construction is another issue that needs to be considered. Reportedly, the plasmid microinjected into the cytoplasm can be transported into the nucleus by active transport through binding to a transcription factor with a nuclear localization signal sequence when the plasmid is carrying specifically designed sequences, such as the SV40 enhancer sequence^60–62^.

Another difference may be the cell type. Different cell types are considered to have different components of NE and NPC^63,64^. This difference may be related to the nuclear translocation of foreign DNA, although it remains unclear. Alternatively, different cell types may suggest differences in NE fragility. In fact, transient NE ruptures have been reported in cells lacking lamin A and in cancer cells defective in NE^65,66^. Notably, premitotic exogenous protein expression was observed in the cells with transiently ruptured NE (Fig. 6a) and with the NE depleted with emerin (Fig. 6b), indicating that plasmids can enter the nucleus in the presence of the deficient NE with reduced barrier function. BAF has been reported to play a role in repairing NE rupture^67^; however, the involvement of BAF in the nuclear entry of plasmids during transient NE rupture remains unclear.

It has been reported that most plasmids form clusters in the cytosol after endosomal escape, regardless of the transfection method; moreover, the clusters are relatively immobile during interphase^17^. The clusters remaining at metaphase are asymmetrically partitioned with a bias toward the younger centrosomes in dividing cells^17^. We also found similar plasmid clusters in the cytosol. However, most of them were dispersed at the beginning of mitosis to smaller puncta. The small puncta were scattered throughout the cell and distributed to daughter cells (Fig. 2a and Supplementary Movies 1, 3), whereas the large puncta were retained in one cell, as reported previously^17^. Despite the unclear relationship between the size of the DNA punctum and gene expression, our time-lapse analysis suggests that the presence of small puncta during mitosis leads to gene expression (Supplementary Fig. 2a, b). In addition, CLEM and iCLEM analyses identified small puncta attached to the telophase chromosome (Figs. 3 and 4). Furthermore, we showed that gene expression was delayed by the depletion of BAF (Fig. 5c–e). As BAF is required for NE formation during telophase^25,27^ and as depletion of BAF delays NE formation^27^, these results together support the idea that NE reformation at telophase is important for the incorporation of the plasmid into the nucleus.

Transfected DNA in the cytosol activates DNA sensor signaling pathways^6,7,68^. Binding of DNA sensors to the cytosolic DNA is hypothesized to be a defense response against infection. Many DNA sensor proteins have been identified, including cyclic GMP-AMP synthase (cGAS)^69^ and their common signaling adaptor stimulator of interferon (IFN) gene (STING)^70^, DNA-dependent activator of interferon regulatory factors/Z-DNA-binding protein 1 (DAI/ZBP1)^71^, interferon activated gene 204 (p204)^72^, DEAD box helicase 60 (DDX60)^73^, and BAF^8,30^. DAI/ZBP1 and p204 have been reported to bind cytosolic plasmid DNA within 15 min, whereas DDX60 does not^74^. BAF binds the cytosolic DNA within seconds^8^ and plays a role in assembling the NE-like membrane around the plasmid DNA within approximately 10 min^8^. This BAF-dependent NE assembly protects foreign DNA from autophagy^8,30^. Although BAF is expected to function as a defense against viral infection^29,75,76^, it unexpectedly assists in the integration of the human immunodeficiency virus (HIV) genome into the host genome by preventing the suicidal autointegration to the viral genome as named after barrier-to-autointegration factor^21,22^. In addition, a recent report showed that BAF protects against the cGAS-STING response to prevent innate immune activation^77,78^. Collectively, these data suggest that BAF can suppress the innate immune response and act to safely carry the cytosolic DNA to the next cell-cycle stages by wrapping it in the NE-like membrane.

In summary, our data suggest that NE acts as a barrier for foreign DNA to enter the nucleus, and that foreign DNA is incorporated into the nucleus when the NE is being reassembled in telophase. Our findings provide important insights not only for understanding cellular responses during DNA transfection, but also for the development of transfection reagents and methods.

## Methods

### Plasmids

We constructed the plasmid carrying a *lacO* repeat sequence and a sequence expressing RFP driven by the EF1α promoter (pLacO-pEF1α-RFP) as follows: First, to generate the mRFP-C1 vector, the DNA fragment encoding mRFP was amplified by PCR from the mRFP-pRSETB vector using the primer set mRFP-FWD3-Nhe (5′-GAGCTAGCATGGCCTCCTCCGAGGACGTCA-3′) and mRFP-REV2-Bgl (5′-TAAGATCTGGCGCCGGTGGAGTGGCGGCCC-3′), and the PCR product was inserted into the pEGFP-C1 vector (Clontech, Takara Bio Inc., Kusatsu, Japan) at the *Nhe*I and *Bgl*II sites. The resulting plasmid is called the mRFP-C1 vector. Next, to generate the pEF1α-mRFP plasmid, the DNA fragment encoding the EF1α-promoter region was amplified by PCR from the pBOS-H2B-GFP vector (Stratagene, La Jolla, CA) using the primers EF1α-prom.FWD-Ase (5′-GGGATTAATCGTGAGGCTCCGGTGCCCGTCAGTGG-3′) and EF1α-prom.REV-Nhe (5′-GGGCTAGCCTCACGACACCTGAAATGGAAGAAAAAAACTTTG-3′), and the PCR product was inserted into the mRFP-C1 vector at the *Ase*I and *Nhe*I sites. The resulting plasmid is called the pEF1α-RFP plasmid. Then, the pEF1α-RFP plasmid was digested with *Sal*I, then circularized with a ligation kit (Ligation High Ver.2, Toyobo Co., Osaka, Japan) after filling the gaps with *E. coli* DNA polI Klenow fragment (Takara Bio Inc., Kusatsu, Japan) to generate the pEF1α-mRFP-C1-ΔSalI vector. The pEF1α-mRFP-C1-ΔSalI vector was inserted with the sequence of TAGTCGAC to generate a new *Sal*I site in front of the EF1α promoter region to generate the SalI-pEF1α-mRFP-C1 vector. Finally, to generate pLacO-pEF1α-RFP, the DNA fragment containing a *LacO*-repeat sequence was obtained by digestion of the pSV-dhfr DNA plasmid, then inserted into the SalI-pEF1α-mRFP-C1 vector at its *Sal*I site using Ligation High Ver.2 (Toyobo Co., Osaka, Japan).

To construct the plasmid expressing EGFP-fused LacI (pGFP-LacI), EGFP was amplified by PCR from the pEGFP-C1 vector (Clontech, Takara Bio Inc., Kusatsu, Japan) using the primers 5′-TGTGACCGGGCGCCTACTATGGTGAGCAAGGGCGAGGAGCT-3′ and 5′-CGAATTCGCTAGCTCTAGACTTGTACAGCTCGTCCATGC-3′, and the PCR product was inserted into the PB-EF1α-MCS-IRES-Neo vector (PB533A-2, System Biosciences, Mountain View, CA, USA) using *Xba*I sites to generate the PB-EF1α-EGFP-MCS-IRES-Neo vector. Next, DNA encoding LacI was amplified from the MK9-38/pYC19 vector using the primers (5′-ATTCGAATTTAAATCGGATATGGTGAAACCAGTAACGTTATA-3′ and 5′-CTCAGCGGCCGCGGATCTTACAGCTGCATTAATGAATCGGCC-3′), and the PCR product was inserted into the PB-EF1α-EGFP-MCS-IRES-Neo vector using a *Bam*HI site to generate the plasmid expressing GFP-LacI.

### Cell strains and culture

HeLa and U2OS cells were obtained from the Riken Cell Bank (RCB007, Tsukuba, Japan) and the Health Protection Agency (London, UK), respectively. HeLa cells were maintained in DMEM containing 10% calf serum (CS) and U2OS cells were in DMEM containing 10% fetal bovine serum (FBS). To generate HeLa cells stably expressing GFP-LacI (HeLa/GFP-LacI), they were transfected with plasmids encoding GFP-LacI using Effectene (Qiagen, Hilden, Germany), according to the manufacturer’s instructions. Cells were selected with 800 μg/ml Geneticin (11811-031, Life Technologies, Thermo Fisher Scientific, Tokyo, Japan). Several clones were isolated from the survivors and their expression levels were evaluated using Western blotting (WB) analysis. The selected clones were maintained in DMEM containing 10% FBS and 200 μg/ml Geneticin (11811-031, Life Technologies, Thermo Fisher Scientific, Tokyo, Japan). We established *LEMD2* mutant cell lines (HeLa/mClover3-mAID-Lem2) expressing an mClover3-mAID-Lem2 protein as the sole Lem2 protein as described below (see section Depletion of NE proteins in Methods).

### Antibodies

Primary mouse monoclonal antibodies included anti-BAF for IF (1:100 dilution, BANF1, Cat No. H00008815-M01, Lot No. 6132-S3, Abnova, Taipei, Taiwan) in Fig. 1b, anti-emerin for IF (1:300 dilution, H-12, sc-25284, Santa Cruz) in Supplementary Fig. 1a, anti-calnexin (CNX) for IF (1:100 dilution, 610523, BD Bioscience) in Supplementary Fig. 1a, anti-early endosome antigen 1 (EEA1) for IF (1:100 dilution, 610456, BD Bioscience) in Supplementary Fig. 1a, anti-phosphorylated histone H3 at the 10th serine residue (1:2000 dilution, H3-S10P)^79^ for WB in Supplementary Fig. 1d, anti-β-actin for WB (ab8224, Lot No. GR14272-3, Abcam) in Supplementary Figs. 1d (1:1000 dilution), 5a, b (1:5000 dilution), anti-α-tubulin (1:5000 dilution, clone DM1A, Cat No. T9026, Lot No. 017H4838, Sigma-Aldrich) in Figs. 5d and 6c, and anti-GFP for WB (1:2000, JL-8, Cat No. 632381, Lot No. 1004037, Clontech, USA) in Supplementary Fig. 5a.

Primary rabbit polyclonal antibodies included anti-GFP antibody for iCLEM (1:200 dilution, Cat No. 600-401-215, Lot No. 24437, Rockland, Limerick, PA, USA), anti-emerin for IF (1:300 dilution, ED1^80^) in Figs. 1b, 2b, c, and Supplementary Fig. 2c, and for WB (1:3000 dilution, ED1) in Fig. 6e, anti-BAF for WB (1:1000, PU38143)^81^ in Fig. 5d and Supplementary Fig. 6a, anti-Lem2 for IF (1:100 dilution, LEMD2, HPA017340, Lot No. B96759, Atlas Antibodies, Bromma, Sweden) in Supplementary Fig. 1a, and for WB (1:1000 dilution, LEMD2, HPA017340, Lot No. B96759) in Supplementary Fig. 5a, b, anti-lamin B receptor (LBR) for IF (1:300 dilution, 1398-1, Epitomics) in Supplementary Fig. 1a, anti-lamin B1 for IF (1:2000 dilution, ab16048, Abcam) in Supplementary Fig. 1a, and anti-RFP for WB (1:1000 dilution, PM005, MBL) in Supplementary Fig. 1d.

Alexa594-conjugated secondary antibodies against mouse IgG (1:1000 dilution, A11037, Thermo Fisher Scientific) and rabbit IgG (1:1000 dilution, A11032, Thermo Fisher Scientific) were used to detect the primary antibodies for iCLEM. Horseradish peroxidase-conjugated anti-mouse IgG (1:2000 dilution, NA9310V, GE Healthcare) and anti-rabbit IgG (1:2000 dilution, NA9340V, GE Healthcare) were used for WB.

### Transfection

Cells were seeded on 35-mm glass-bottom culture dishes (MatTek, MA, USA) at a concentration of 2 × 10^5^ per dish one day before imaging. The cells were incubated for 4 h with Effectene transfection reagent (Qiagen) containing 0.5 μg/dish DNA plasmid. Hoechst 33342 was added to a final concentration of 100 ng/ml to stain DNA 15 min before the removal of the transfection reagent. After the removal of the transfection reagents, the cells were washed with the prewarmed culture medium twice, and subjected to time-lapse live-cell imaging as described below. For immunofluorescence staining, CLEM and iCLEM analyses, the cells were fixed with the fixative as described in the corresponding Method sections.

### Time-lapse live-cell imaging

Cells were transfected with plasmids using Effectene as described above. After transfection reagents were removed, the prewarmed observation medium (phenol red-free DMEM containing 10% FBS and 25 mM Hepes buffer, pH 7.3) was added to the cells. Then, the cells were subjected to time-lapse live-cell imaging. Images were obtained every 10 min (1 μm × 7 *z*-stacks for a single time point unless otherwise specified) using an oil-immersion objective lens UApo/340 (×40, NA = 1.35, Olympus, Tokyo, Japan) on a DeltaVision fluorescence microscope system (GE Healthcare Japan, Tokyo, Japan) in a temperature-controlled (37 °C) room or through a water-immersion objective lens C-Apo40 (×40, NA = 1.2, Carl Zeiss, Jena, Germany) on a confocal fluorescence microscope system LSM-880 (Carl Zeiss), maintained at 37 °C using a stage warmer.

### Immunofluorescence staining

HeLa/GFP-LacI cells were transfected with plasmid using Effectene as described above. After removal of the transfection reagent, cells were fixed with a fixative (3.7% formaldehyde and 0.2% glutaraldehyde in PBS) for 10 min at room temperature (RT, approximately 26 °C), and permeabilized with 0.1% Triton X-100 in PBS for 5 min at RT. The cells were treated twice with 0.1% sodium borohydride in PBS for 15 min at RT, and incubated with 1% BSA in PBS for 1 h at RT^25^. Then, the cells were incubated with anti-BAF (1:100 dilution) or anti-emerin antibodies (1:300 dilution)^25,81^. Anti-rabbit IgG-conjugated Alexa594 (1:500 dilution) was used as the secondary antibody. Fluorescence images were obtained using an Olympus oil-immersion objective lens PLAPON60xOSC (NA = 1.40) on a DeltaVision microscope system. *Z*-stacks of images (typically 20-30 focal planes at 0.5 μm intervals) were obtained and deconvoluted using software provided with the microscope system, SoftWorx.

### Super-resolution microscopy

HeLa/GFP-LacI cells were transfected with plasmid using Effectene as described above. After removal of the transfection reagent, cells were fixed with a fixative (3.7% formaldehyde and 0.2% glutaraldehyde in PBS). Then, cells were observed using an oil-immersion objective lens Plan-Apochromat ×63/1.40 M27 (Carl Zeiss) and immersion oil (#518 F, Carl Zeiss) on an LSM880 Airyscan microscope system (Carl Zeiss) equipped with at room temperature (RT, approximately 26 °C). Pinhole size was set to 384 µm. Hoechst and GFP were excited with 405 and 488 nm laser, respectively. The fluorescent signals for Hoechst and GFP were collected using the following beam splitters and bandpass filters: MBS-405 and BP 420-480 for Hoechst and MBS488/561 and BP 495-550 for GFP. Images were acquired at 988 pixels each with 0.037 µm/pixel. A *z*-stack image set was acquired for 133 focal planes with 0.159 µm intervals. After processing for super-resolution image, brightness and contrast were changed for better visibility in Zen 2.3 SP1 software without changing gamma.

### Correlative light and electron microscopy (CLEM)

HeLa/GFP-LacI cells were transfected with plasmid using Effectene as described above. After removal of the transfection reagent, cells were fixed with 2.5% glutaraldehyde for 1 h. CLEM was performed with some modifications of Live CLEM^27^. Briefly, after washing with PBS, the telophase cells 8–10 min after metaphase to anaphase transition were selected by morphology, then subjected to fluorescence microscopy (FM) to obtain 3D images (typically 40–60 focal planes at 0.2 μm intervals) using a low-chromatic-aberration oil-immersion lens PLAPON60xOSC (NA = 1.40, Olympus) on a DeltaVision microscope system. The images were deconvoluted to remove out-of-focus images using software provided with the microscope system. Samples were post-fixed with 1% OsO_4_ (3002, Nisshin EM, Tokyo, Japan), stained with 2% uranyl acetate (8473-1 M, Merck, Darmstadt, Germany), and then embedded in Epon812 (T024, TAAB, England). Cells observed by FM were identified in the block. Ultrathin sections of 80 nm were prepared and further stained with 2% uranyl acetate, followed by a commercial ready-to-use solution of lead citrate (18-0875-2, Sigma-Aldrich, St. Louis, MO, USA). Electron microscopy (EM) images were acquired using a JEM-1400 electron microscope (80 kV; JEOL, Tokyo, Japan). To correlate the FM and EM images, we selected the FM image corresponding to the EM image from the 3D FM images by eye using criteria including nuclear shapes and areas of bead cross-sections.

### Immuno-CLEM (iCLEM)

HeLa/GFP-LacI cells were transfected for 4 h with 0.2 μg/dish pLacO-pEF1α-RFP using Effectene as described above. After removal of transfection reagents, cells were fixed with a mixture of 3.7% formaldehyde and 0.2% glutaraldehyde for 15 min. After washing with PBS, cells were permeabilized with 0.01% Triton X-100 in PBS for 5 min at RT (26 °C), treated twice with 0.1% sodium borohydride in PBS for 15 min, and washed twice with 1% BSA in PBS for 1 h. Cells were then incubated with anti-GFP antibody (1:500 dilution in 1% BSA in PBS, 600-401-215, lot. No. 35459, Rockland). After extensive washing with PBS (10 min × 12 times), cells were incubated with a secondary Alexa594-nanogold-anti-rabbit antibody (1:200 dilution in 1% BSA in PBS, Nanoprobes, Yaphank, NY, USA) for 3 h at RT. A *z*-stack of fluorescence images (typically 40–60 focal planes at 0.2-μm intervals) were acquired for cells of interest using a low-chromatic-aberration PLAPON60xOSC (NA = 1.40) objective on a DeltaVision microscope system as described above. Then, cells were fixed with 1% glutaraldehyde for 1 h and washed with PBS three times. After washing twice with 100 mM Lysine-HCl/PB (pH 7.4) for 10 min, cells were subjected to silver enhancement as follows^27^: cells were washed with PBS, incubated with 50 mM HEPES (pH 5.8) three times, and washed with distilled water. Cells were then treated with silver-enhancing reagent at 25 °C for 5 min and washed with distilled water three times. Samples were post-fixed with 1% OsO_4_ (3002, Nisshin EM, Tokyo, Japan), stained with 2% uranyl acetate (8473-1 M, Merck, Darmstadt, Germany), and then embedded in Epon812 (T024, TAAB, England). Cells observed by FM were identified in the block. Ultrathin sections of 80 nm were prepared and further stained with 2% uranyl acetate, followed by a commercial ready-to-use solution of lead citrate (18-0875-2, Sigma-Aldrich, St. Louis, MO, USA). Electron microscopy (EM) images were acquired using a JEM-1400 electron microscope (80 kV; JEOL, Tokyo, Japan). To correlate the FM and EM images, we selected FM image corresponding to EM images from the 3D FM images by eye, using criteria including nuclear shape and area of bead cross-section in each image.

### Depletion of NE proteins

To deplete BAF, HeLa cells or HeLa cells stably expressing GFP-BAF were treated with *BAFN1* siRNA [AAGAAGCTGGAGGAAAGGGGT, Qiagen]^27^ using RNAiMax (ThermoFisher Scientific). To deplete emerin, HeLa cells were treated with *EMR* siRNA [AACCGTGCTCCTGGGGCTGGG, Qiagen]^82^ using RNAiMax. Luciferase siRNA (Qiagen) was used as the negative control.

The AID-induced protein degradation system was used to deplete Lem2^50^. The plasmid pAID1.2-EF1a-NmClover3-mAID was developed from pAID1.2-EF1a-NGFP-mAID (Addgene plasmid #140607). The cDNA sequence of Lem2 was amplified by PCR and cloned into the pAID1.2-EF1a-NmClover3-mAID vector at the *Eco*RV site (pAID1.2-EF1a-NmClover3-hsLem2) using the In-Fusion HD Cloning Kit (TaKaRa, Kusatsu, Shiga, Japan). The pX330 plasmid targeting the human *LEMD2* gene (hsLEMD2-pX330) was constructed as follows: The sequence 5′-GACTTACTCACCAGCTTGGATGG-3′ at the intron-exon boundary of the 3′ end of exon 3 of the human *LEMD2* gene was selected as a target for CRISPR/Cas9-mediated gene editing. These plasmids were cotransfected with pAID EF1a linearized in pX330 (plasmid #140610, Addgene) into HeLa cells. After selection in 8 µg/ml blasticidin S (029-18701, Wako, Osaka, Japan), several clones were picked; their expression levels of endogenous and mClover3-mAID-tagged Lem2 were evaluated by WB using anti-Lem2 (1:1000 dilution) and anti-GFP antibodies (1:2000 dilution), respectively. We used an anti-GFP antibody to detect mClover3-mAID-Lem2 because the anti-GFP antibody could cross-react with mClover3. AID-mediated protein degradation was induced by adding final 0.5 mM indole-3-acetic acid (IAA).

### Mitotic arrest with nocodazole

HeLa/GFP-LacI cells were transfected with the plasmid (pLacO-pEF1α-RFP) using Effectene as described above. After washing with culture medium twice to remove the transfection reagents, the cells were incubated with the culture medium for 18 h in the presence of 100 μg/ml nocodazole (M1404, Sigma-Aldrich, St. Louis, MO), a microtubule inhibitor, or dimethyl sulfoxide (DMSO) as a control. To release the cells from cell-cycle arrest by nocodazole, the cells were washed twice with a prewarmed culture medium not containing nocodazole and incubated in the medium for another 24 h.

### Western blotting (WB)

Cells were collected and lysed in ice-cold isotonic buffer (20 mM Tris-HCl, pH 7.5, 2 mM MgCl_2_, 150 mM NaCl, 0.5% NP-40, Protease Inhibitor Cocktail) (final 1 × 10^7^ cell/ml). Equal amounts of 2× SDS-PAGE sample buffer (without DTT) were added to the lysates. After sonication, the lysates were boiled for 5 min. DTT (freshly prepared from powder) was added to the samples at a final concentration of 20 mM. Ten micrograms of total protein from the cell extract were loaded into each lane of an SDS 10–20% gradient polyacrylamide gel or 15% polyacrylamide gel. After electrophoresis, proteins were transferred to PVDF membranes using a semi-dry blotting system (Atto Corp., Tokyo, Japan) and stained using the ImmunoStar LD (296-69901, Fujifilm, Osaka, Japan) or Zeta (295-72404, Fujifilm).

### NE rupture

HeLa/GFP-LacI cells were transfected using the transfection reagent Effectene (Qiagen) with 0.3 μg/dish of pLacO-pEF1α-RFP for 3 h. During this incubation, Hoechst 33342 was added to a final concentration of 100 ng/ml and incubated for 15 min to stain DNA. After removal of the transfection mixture, the culture medium was replaced with fresh DMEM containing 10% FBS and then subjected to fluorescence live-cell imaging. Cells in the glass-bottom culture dish were placed on a stage of the DeltaVision fluorescence microscope in a temperature-controlled room (37 °C). Cells of interest, i.e., the cells appearing to be undergoing G1 phase, were selected based on the morphology observed via bright-field microscopy; their NEs were disrupted using the tip of a microinjection needle (Femtotips, Eppendorf, No. 5242952008 Eppendorf, Hamburg, Germany) attached to a microinjector (microinjector 5242 and micromanipulator 5171, Eppendorf). To confirm NE rupture, cells were concomitantly microinjected with 0.5 μg/μl FITC-dextran (MW, 10 kDa) as an injection marker. Fluorescence images were obtained at 10–15 min intervals for 4 h.

### Statistics and reproducibility

The timing of RFP expression was determined in individual cells by time-lapse live-cell imaging. The number of cells examined in each analysis (Figs. 1d, 5b, e and Supplementary Figs. 1e, 6b, e) was over 100 (range of *n* = 125–594). The median values of the timing for each analysis were calculated using Microsoft Excel (version 16.54). The cell numbers and the median values are shown in each figure.

### Reporting summary

Further information on research design is available in the Nature Research Reporting Summary linked to this article.

## Supplementary information

Supplementary Information

Description of Additional Supplementary Files

Supplementary Movie 1

Supplementary Movie 2

Supplementary Movie 3

Supplementary Movie 4

Supplementary Movie 5

Supplementary Data 1

Reporting Summary

## Data Availability

The authors declare that the data supporting the findings of this study are available within the paper and Supplementary Information. The plasmids generated in this paper are deposited in Addgene, and available as pLacO-pEF1α-RFP (Addgene plasmid #179507) and pGFP-LacI (Addgene plasmid #179510), hsLEMD2-pX330 (Addgene plasmid #179511), and pAID1.2-EF1a-NmClover3-hsLem2 (Addgene plasmid #179524). Further data are available from the corresponding authors upon request.
