# Supplementary material for: Transfected plasmid DNA is incorporated into the nucleus via nuclear envelope reformation at telophase
Source: Commun Biol. 2022 Jan 20;5:78. doi: 10.1038/s42003-022-03021-8 (PMC8776997; doi:10.1038/s42003-022-03021-8)
Supplement: Supplementary file 1 — Supplementary Information [file 42003_2022_3021_MOESM1_ESM.pdf]

Supplementary Information to:

Transfected plasmid DNA is incorporated into the nucleus via nuclear envelope reformation at telophase

Index:

Supplementary Figure 1.	2
Supplementary Figure 2.	4
Supplementary Figure 3.	6
Supplementary Figure 4.	8
Supplementary Figure 5.	10
Supplementary Figure 6.	12

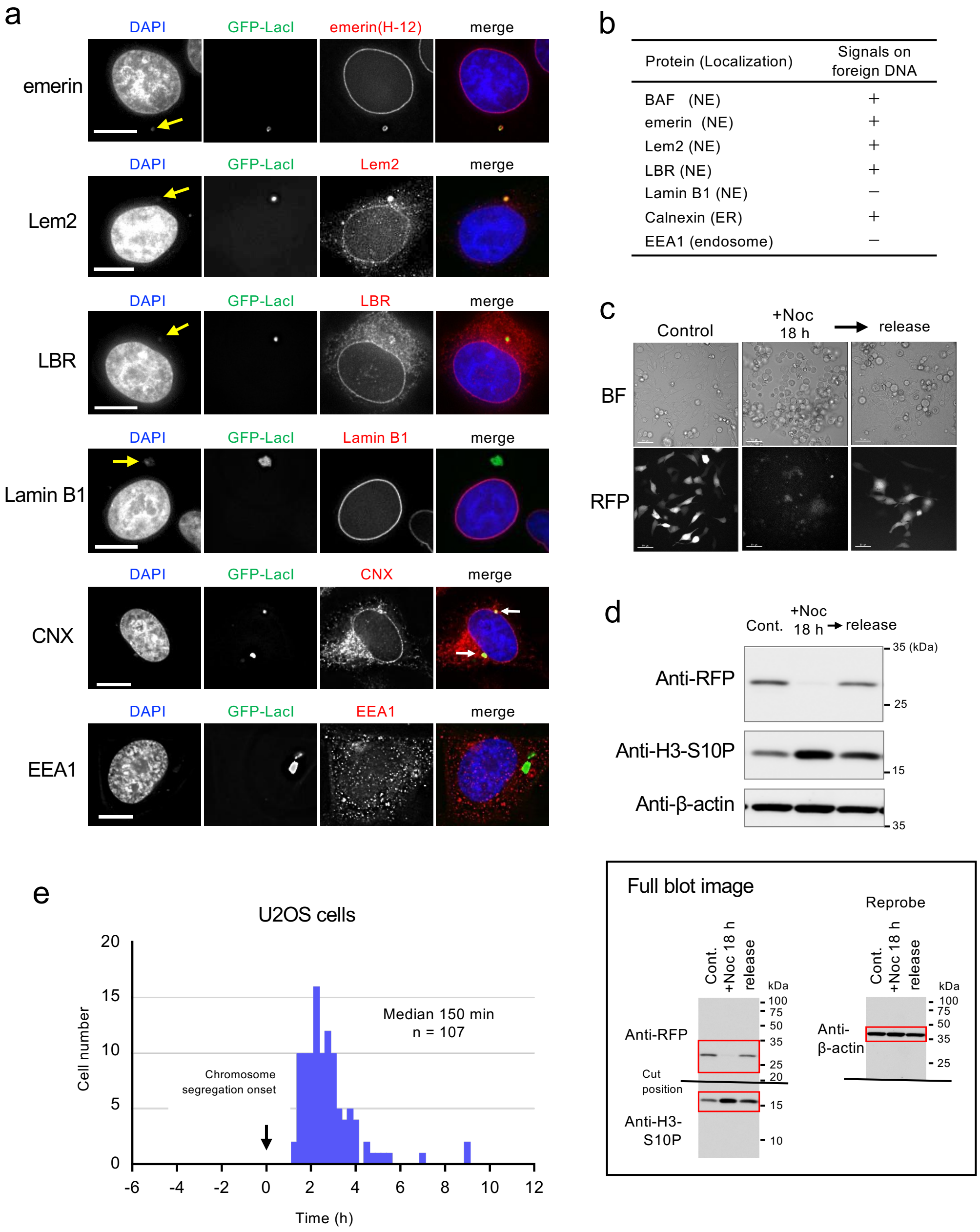

Supplementary Figure 1. Protein components around the transfected DNA and gene expression from the transfected DNA.

a. HeLa/GFP-LacI cells were transfected with the pLacO-pEF1α-RFP plasmid (0.5 μg/dish) using Effectene transfection reagent for 4 h. Immediately after transfection, cells were fixed and stained using specific antibodies against emerin, Lem2, lamin B receptor (LBR), lamin B1, calnexin (CNX) and early endosome antigen 1 (EEA1). Anti-emerin antibody (H-12, commercially available monoclonal anti-emerin antibody), different from one used in Fig. 1b (ED1, house-made rabbit polyclonal anti-emerin antibody), was

used here. DNA was stained with DAPI. Colors represent DAPI (blue), GFP-LacI (green), and the indicated antigen proteins (red) in merged image. Large arrows represent the positions of the cytoplasmic DAPI puncta colocalized with GFP-LacI puncta. Small arrows indicate the positions of CNX positive GFP-LacI puncta. Bar, 10 μm.

b. A summary table of the results in (**a**). The + and – marks indicate positive and negative, respectively, in colocalization of each protein with the GFP-LacI puncta.

c. Effect of mitotic arrest with nocodazole on exogenous RFP expression. HeLa/GFP-LacI cells were transfected with the pLacO-pEF1α-RFP plasmid using Effectene. After transfection, the cells were cultured without (control) or with 100 μg/ml nocodazole (+ Noc) for 18 h. For releasing the cells from the cell cycle arrest by nocodazole, cells were washed twice with the culture medium not containing nocodazole and incubated in the medium for 24 h (release). BF and RFP indicate bright field and RFP fluorescence images, respectively.

d. Western blotting (WB) analysis of the lysates from the cells obtained in (**c**). WB was performed as described in Materials and Methods. RFP expression was determined by anti-RFP antibody (top panel). Histone H3 phosphorylation at the 10th serine residue (H3-S10P) was determined as a mitotic indicator (middle panel). The level of H3-S10P was increased by the addition of nocodazole and decreased by the removal of the drug, indicating that nocodazole effectively arrested the cells at mitosis. β-actin was detected as a loading control (bottom panel). Molecular size markers are indicated in the right. The uncropped/unedited blot images and molecular size markers are displayed in the black square frame. The red frames are cropped regions.

e. U2OS cells were transfected with the pEF1α-RFP plasmid (0.5 μg/dish) using Effectene transfection reagent for 4 h. DNA was stained with Hoechst 33342 during transfection. Immediately after transfection, time-lapse live-cell images for each wavelength were acquired at 10 min intervals (2 μm interval × 5 z-stacks for each time point) for 18 h through an oil immersion objective lens UApo 40× (NA = 1.35, Olympus) using the DeltaVision microscope in a temperature-controlled room (37°C). The timing of RFP expression from the transfected DNA was determined by the appearance of RFP signals in individual living cells, and the timing of chromosome segregation was determined for each of the individual cells. X-axis represents the time of RFP expression in hours when the timing of chromosome segregation is set to zero for each cell. Y-axis is the number of cells that expressed RFP at that time. The total number of cells plotted was 107. The median time of RFP expression was 150 min after the onset of mitotic chromosome segregation.

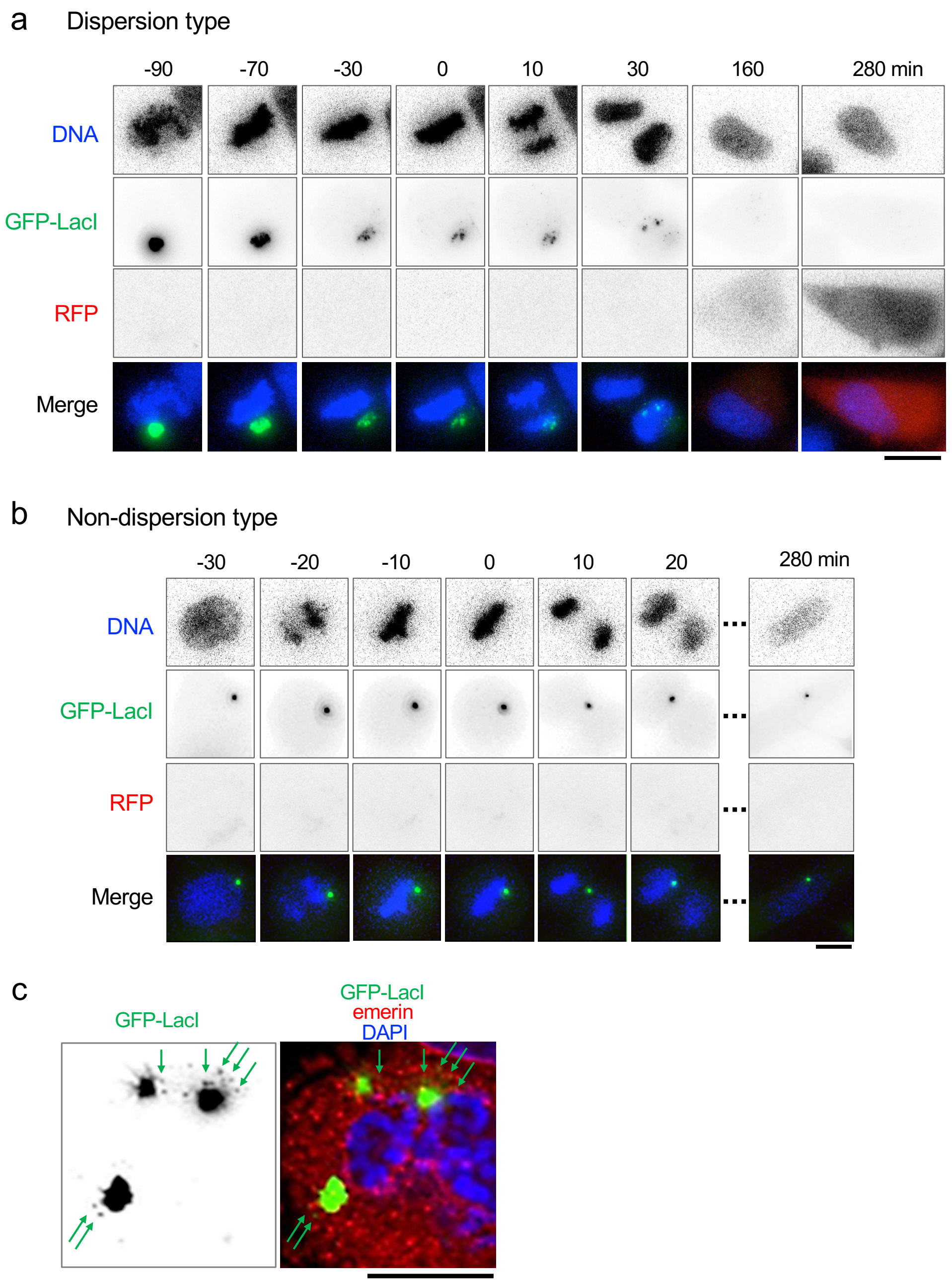

Supplementary Figure 2. Dynamic behavior of transfected DNA during mitosis.
a. A representative example of a cell showing dispersion of GFP-LacI puncta during mitosis. HeLa/GFP-LacI cells were transfected with pLacO-pEF1α-RFP. DNA was stained with Hoechst 33342 during transfection. After the transfection reagent was replaced with the culture medium, time-lapse images for each wavelength were acquired at 10 min intervals (1 μm interval × 7 z-stacks for each time point). Time 0 represents the onset of chromosome segregation. Black-and-white inverted images are shown for each wavelength. Merged images, DNA (Hoechst 33342, blue), GFP-LacI (green), and RFP

(red). Bar, 10 μm. RFP expression was observed 160 min after the onset of chromosome segregation.

b. A representative example of a cell showing non-dispersion of GFP-LacI puncta during mitosis. Sample preparation and image acquisition were same as in (**a**). Time 0 represents the onset of chromosome segregation. Black-and-white inverted images are shown for each wavelength. Merged images, DNA (Hoechst 33342, blue), GFP-LacI (green), and RFP (red). Bar, 10 μm. No RFP expression was observed until 280 min after the onset of chromosome segregation.

c. Enlarged images of the lower panels in Fig. 2b. HeLa/GFP-LacI cells transfected with pLacO-pEF1α-RFP, fixed, and immuno-stained using anti-emerin antibody (ED1). DNA was stained with DAPI. Left, GFP-LacI (black-and-white inversion). Right, merged images. GFP-Lac1 (green), emerin (red), and DAP1 (blue) in the merged image. Arrows, the small puncta of GFP-LacI. Bar, 10 μm.

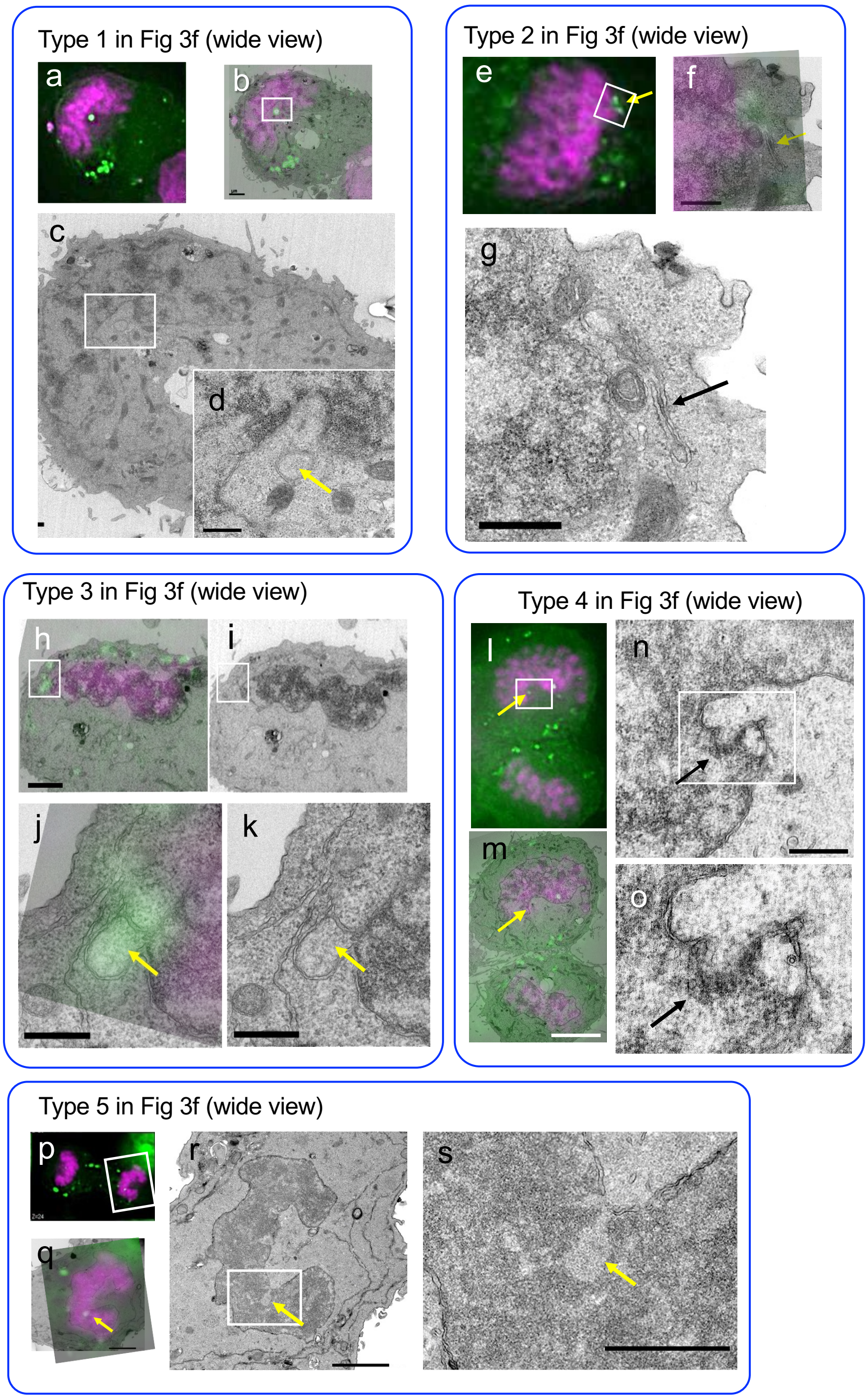

Supplementary Figure 3. CLEM analysis of telophase cells.
Overview images of the same cells shown in Figure 3f. Green and magenta represent GFP-LacI and DAPI signals, respectively. Arrows indicate the position of the small puncta of GFP-LacI.

a-d: Type 1 (cytoplasmic circle) localization of transfected DNA. **a.** Fluorescence image. **b.** Merged image of fluorescence micrograph and electron micrograph. Bar, 2 μm. **c.** Enlarged electron microscopy image of (**b**). **d.** Higher magnification of the boxed region in (**b**) and (**c**). Bar, 500 nm.

e-g: Type 2 (sandwich) localization of transfected DNA. **e.** Fluorescence image. **f.** Merged image of fluorescence image and electron micrograph. **g.** Electron micrograph of the boxed region in (**e**). Bar, 2 μm.

h-k: Type 3 (membrane fusion) localization of transfected DNA. **h.** Merged image of fluorescence image and electron micrograph. Bar, 2 μm. **i.** Electron micrograph. **j.** Higher magnification of the boxed region in (**h**) and (**i**). Bar, 500 nm. **k.** Electron micrograph of (**j**). Bar, 500 nm.

l-o: Type 4 (attached to the chromosome) localization of transfected DNA. **l.** Fluorescence image. **m.** Merged image of fluorescence image and electron micrographs. Bar, 5 μm. **n.** Electron micrograph of the boxed region in (**l**). **o.** Higher magnification of the boxed region in (**n**).

p-s: Type 5 (inside the nucleus) localization of transfected DNA. **p.** Fluorescence image. **q.** Merged image of fluorescence image and electron micrograph in the boxed region in (**p**). **r.** Electron micrograph of the same region as (**q**). Bar, 2 μm. **s.** Higher magnification of the boxed region in (**r**). Bar, 1 μm.

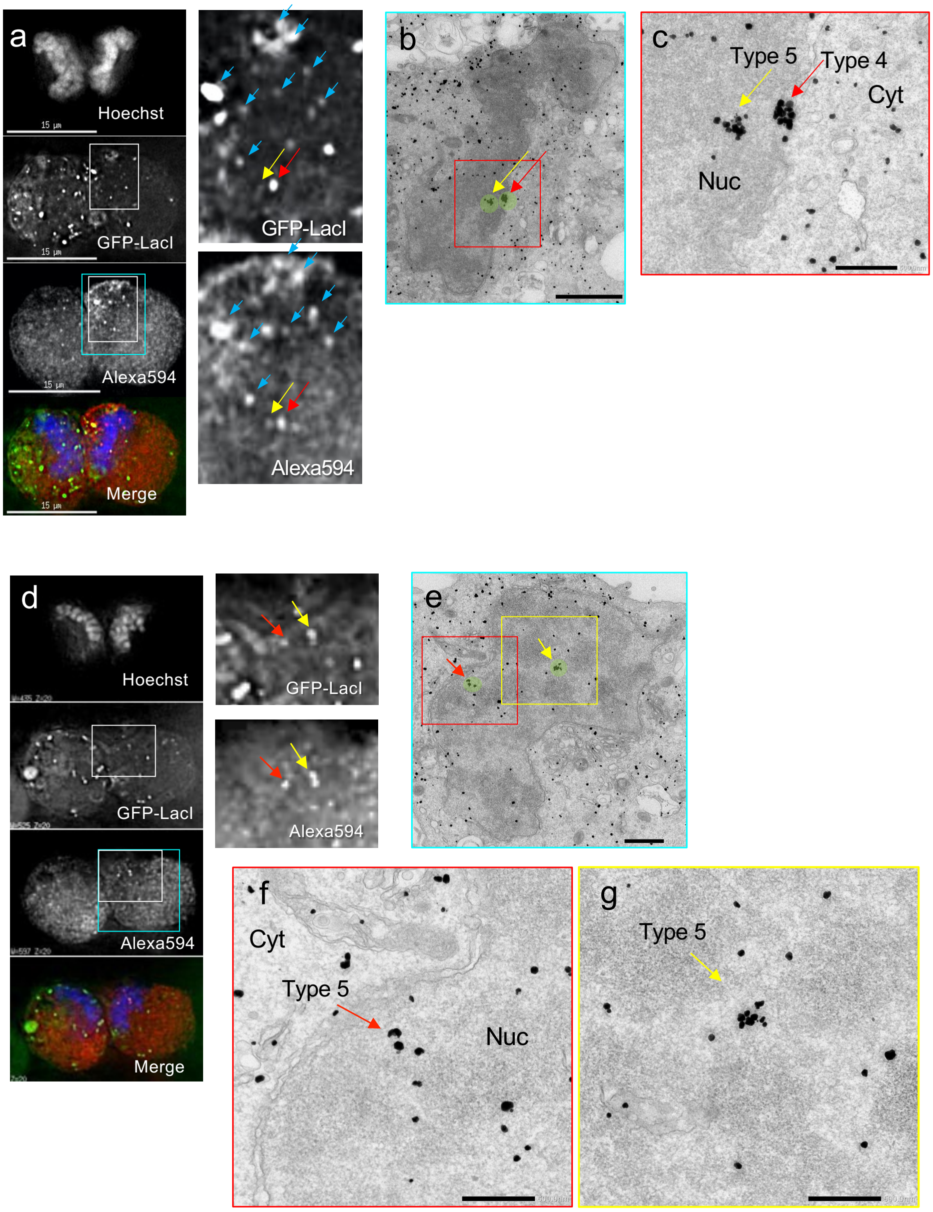

Supplementary Figure 4. Immuno-CLEM analysis of telophase cells

Immuno-CLEM imaging of telophase HeLa/GFP-LacI cells transfected with pLacO-pEF1α-RFP. Cells were fixed with a fixative, and subjected for immuno-CLEM.

a-c: Representative example of Types 4 and 5 puncta. **a.** Left: Overview fluorescence images of DNA puncta near the chromosomal region. Merged images, Hoechst (blue), GFP-LacI (green), and Alexa (red) signals. Yellow and red arrows, puncta indicated in (**b**) and (**c**). Right: Enlarged views of the white-boxed region in the left images. Arrows indicate puncta where both GFP-LacI and Alexa signals are positive. **b.** Electron micrograph of the blue-boxed region in (**a**). The yellow and red arrows indicate the same puncta pointed by the arrows in (**a**). Bar, 2 µm. **c.** Magnified view of the red-boxed region in (**b**). Nuc, and Cyt label nucleus, and cytoplasm, respectively. Bar, 500 nm.

d-g: Another representative example of Type 5 puncta. **d.** Left: Overview fluorescence images of DNA puncta near the chromosomal region. Merged images, Hoechst (blue), GFP-LacI (green), and Alexa (red) signals. Yellow and red arrows, puncta indicated in (b) and (c). Yellow and red arrows, puncta indicated in (**d**)-(**g**). Right: Enlarged views of the white-boxed region in the left images. **e.** Electron micrograph of the blue-boxed region in (**d**). Bar, 1 μm. **f.** Magnified view of the red-boxed region in (**e**). Bar, 500 nm. **g.** Magnified view of the yellow-boxed region in (**e**). Nuc, and Cyt label nucleus, and cytoplasm, respectively. Bar, 500 nm.

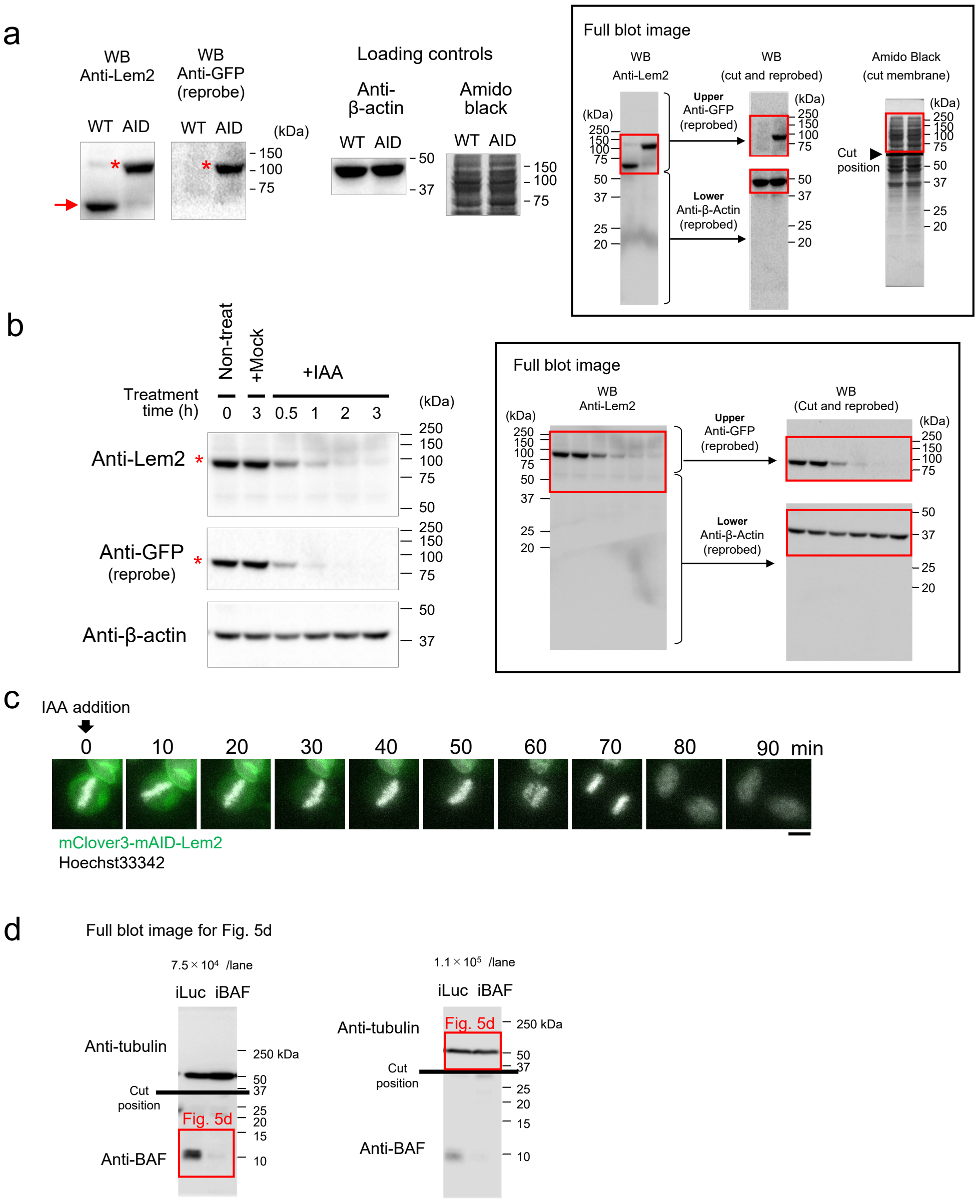

Supplementary Figure 5. Evaluation of the HeLa/mClover3-mAID-Lem2 cell line.

a. Western blotting of lysates of parental HeLa cells (WT) and HeLa/mClover3-mAID-Lem2 (AID). Leftmost panel: Lem2 (WT) and mClover3-mAID-Lem2 (AID) detected by anti-Lem2 antibody as indicated by arrow and asterisk, respectively. The same membrane was reproved with anti-GFP-antibody to produce the next panel: mClover3-mAID-Lem2 detected with an anti-GFP antibody, which recognizes mClover3, as indicated by asterisk. Right panels: anti-β-actin and amido black staining as loading controls. The uncropped/unedited blot images and molecular size markers are displayed in the black square frame. The red frames are cropped regions.

b. Western blotting of lysates of HeLa/mClover3-mAID-Lem2 cells treated with IAA. Lysates were obtained from non-treated (Non-treat), mock-treated (+Mock), IAA-treated

(+IAA) cells (from left to right). Time (h) treated with IAA or mock reagent is indicated on top of each lane. mClover3-mAID-Lem2 (asterisk) were detected by anti-Lem2 antibody (top panel). The same membrane was reproved with anti-GFP-antibody to detect mClover3 (middle panel). Anti-β-actin image is shown as loading controls (bottom panel). Molecular size markers are indicated in the right. The uncropped/unedited blot images and molecular size markers are displayed in the black square frame. The red frames are cropped regions.

c. Time-lapse live-cell images of AID-induced depletion of Lem2 in HeLa/mClover3-mAID-Lem2 cells. DNA was stained with Hoechst 33342. Fluorescence images for each wavelength (Hoechst and mClover-mAID-Lem2) were captured every 1 min (2 μm × 5 z-stacks for each time point) using DeltaVision. Maximum intensity projection images of selected time points are shown. IAA was added to the culture medium during time-lapse imaging. Time 0 represents the time when IAA was added (arrow). mClover3-mAID-Lem2 (green) and Hoechst 33342 (white). Bar, 10 μm.

d. The uncropped/unedited blot images and molecular size markers for Fig. 5d. The red frames are cropped regions.

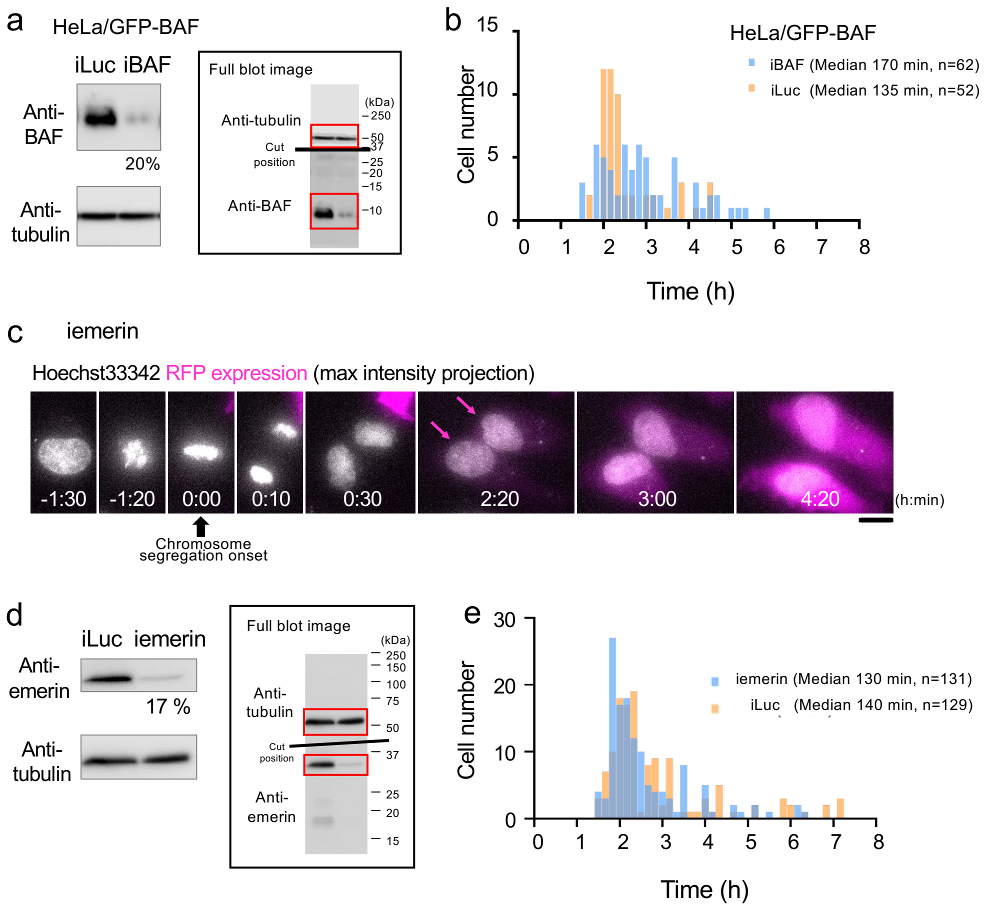

Supplementary Figure 6. Depletion of BAF, but not emerin, delays plasmid gene expression.

a. Western blotting of lysates of HeLa/GFP-BAF cell treated with siRNA targeting *BANF* (iBAF) and control siRNA (iLuc). Protein bands corresponding to endogenous BAF are shown. Anti-tubulin images are shown as loading controls. The uncropped/unedited blot images and molecular size markers are displayed in the black square frame. The red frames are cropped regions.

b. Timing of RFP expression in HeLa/GFP-BAF cells treated with iBAF and iLuc. Cells were treated with siRNA (iBAF or iLuc) and transfected with pEF1α-RFP plasmid. DNA was stained with Hoechst 33342 during transfection. Immediately after transfection, time-lapse images for each wavelength (Hoechst and RFP) were acquired at 10-min intervals (2 μm × 5 z-stacks for a single time-point) using DeltaVision. The timing of RFP expression from the transfected DNA was determined by the appearance of RFP signals in individual living cells, and the timing of chromosome segregation was determined for each of the individual cells. X-axis represents the time of RFP expression in hours when the timing of chromosome segregation is set to zero. Y-axis is the number of cells that

expressed RFP at that time. The total number of cells tested (n) and the median value of RFP expression timing are indicated in the graph.

c. Time-lapse images of transfected HeLa cells treated with siRNA targeting *EMD* (iemerin). HeLa cells were treated with siRNA (iemerin or iLuc), and then were transfected with pEF1α-RFP plasmid. DNA was stained with Hoechst 33342 during transfection. Fluorescence images for each wavelength (Hoechst and RFP) were captured every 10 min (2 μm × 5 z-stacks for each time point). Maximum intensity projection images are shown. Hoechst 33342 (white) and RFP (magenta). Bar, 10 μm.

d. Western blotting of lysates of HeLa cells treated with iemerin and iLuc. Anti-tubulin images are shown as loading controls. The uncropped/unedited blot images and molecular size markers are displayed in the black square frame. The red frames are cropped regions.

e. Timing of RFP expression in HeLa cells treated with iemerin and iLuc. Timings of RFP expression and chromosome segregation was determined for individual cells as described in (**c**). X-axis represents the time of RFP expression in hours when the timing of chromosome segregation is set to zero. Y-axis is the number of cells that expressed RFP at that time. The total number of cells plotted (n) and the median value of RFP expression timing are indicated in the graph.
