# Supplementary material for: Transfected plasmid DNA is incorporated into the nucleus via nuclear envelope reformation at telophase
Source: Commun Biol. 2022 Jan 20;5:78. doi: 10.1038/s42003-022-03021-8 (PMC8776997; doi:10.1038/s42003-022-03021-8)
Supplement: Supplementary file 2 — Description of Additional Supplementary Files [file 42003_2022_3021_MOESM2_ESM.pdf]

Description of Additional Supplementary Files

File name: Supplementary Movie 1.

Description: *Protein expression from transfected DNA imaged by DeltaVision.* HeLa/GFP-LacI cells were transfected with pLacO-pEF1α-RFP, and DNA was stained with Hoechst33342. Time-lapse images for each wavelength were acquired every 10 min (1 µm × 7 z-stacks for each time point) for DNA (blue), GFP-LacI (transfected DNA, green), and RFP (red) using an oil immersion lens UApo 40× (NA = 1.35, Olympus) on a DeltaVision microscope. Maximum intensity projection images are shown. The same cells are shown in Figure 1c. Bar, 10 µm.

File name: Supplementary Movie 2.

Description: *Protein expression from transfected DNA imaged by LSM.* HeLa/GFP-LacI cells were transfected with pLacO-pEF1α-RFP. Time-lapse images for GFP-LacI (green) and RFP (red) were acquired every 10 min (2 µm × 5 z-stacks for each time point) using a water immersion lens C-Apo 40× (NA = 1.2, Carl Zeiss) on a LSM880 confocal microscope. Tiling images were acquired over an area of 1 mm2.

File name: Supplementary Movie 3.

Description: *Behavior of transfected DNA during mitosis.* The same cells are shown in Fig. 2a. HeLa/GFP-LacI cells were transfected with pLacO-pEF1α-RFP, and DNA (red) was stained with Hoechst 33342. Time-lapse images were acquired every 10 min (1 µm × 7 z-stacks for each time point) for DNA (red) and GFP-LacI (green, transfected DNA) using with an oil immersion lens UApo 40× (NA = 1.35, Olympus) on a DeltaVision microscope. The z-stack images were deconvoluted. Maximum intensity projection images for the deconvoluted z-stacks are shown. Dispersion of green puncta (GFP-LacI) during mitosis was observed. Bar, 10 µm.

File name: Supplementary Movie 4.

Description: *Super-resolution three-dimensional images of a cell in metaphase.* The same cells are shown in Figure 2d. HeLa/GFP-LacI cells were transfected with pLacO-pEF1α-RFP. The cells were fixed with a mixture of 3.7% formaldehyde and 0.2% glutaraldehyde, as described in the Methods section. Chromosomes were stained with DAPI. Three-dimensional images (0.2 µm step × 60 z-sections) were acquired using an oil immersion Plan-Apochromat 63× lens (NA = 1.4) on an LSM 880 Airyscan microscope. Super-resolution image processing was performed as described in the Methods section. GFP-LacI (transfected DNA, green) and DAPI (chromosomes, white). Bar, 5 µm.

File name: Supplementary Movie 5.
Description: *AID-induced depletion of mClover3-mAID-Lem2*. The observation field containing the same cell shown in Supplementary Figure 5c. Time-lapse images of the AID-induced degradation of Lem2 in HeLa/mClover3-mAID-Lem2 cells, in which the LEMD gene has been replaced with mClover3-mAID-Lem2 using CRISPR/Cas9. DNA was stained with Hoechst 33342. IAA was added to the culture medium at the beginning of the movie. Fluorescence images for mClover3-mAID-Lem2 (green) and Hoechst 33342 (magenta) were acquired every 1 min (2 µm × 5 z-stacks for each time point) using an oil immersion lens UApo 340 (40×, NA = 1.35, Olympus) on DeltaVision. Maximum intensity projection images are shown.

File name: Supplementary Data 1
Description: Source data for Fig. 1d, 5b, 5e, Supplementary Fig. 1e, 6b, and 6e.
